# Induced fit growth of Ga-based semiconductor thin films for brain-inspired electronics and optoelectronics

**DOI:** 10.1038/s41377-025-02096-2

**Published:** 2026-02-04

**Authors:** Zixu Sa, Kepeng Song, You Meng, Wenfeng Wu, Zhaocong Wang, Pengsheng Li, Jie Zhang, Zeqi Zang, Guangcan Wang, Mingxu Wang, Zhitai Jia, Yang Tan, Weifeng Li, SenPo Yip, Feng Chen, Johnny C. Ho, Zai-xing Yang

**Affiliations:** 1 School of Physics, State Key Laboratory of Crystal Materials, Shandong University, Jinan, China; 2 School of Chemistry and Chemical Engineering, Shandong University, Jinan, China; 3 Department of Materials Science and Engineering, City University of Hong Kong, Hong Kong, China; 4 Institute for Materials Chemistry and Engineering, Kyushu University, Fukuoka, Japan; 5 State Key Laboratory of Terahertz and Millimeter Waves, City University of Hong Kong, Hong Kong, China

**Keywords:** Electronics, photonics and device physics, Photonic devices

## Abstract

Current crystalline thin-film production techniques typically require specific growth substrates, posing significant challenges for their use in flexible electronics and integrated optoelectronics. In response to these challenges, we introduce a novel method called ‘induced fit growth’, inspired by the induced fit theory in molecular biology. This method overcomes the limitations of current techniques by enabling the deposition of Ga-based semiconductor films, including GaSb, GaSe, GaAs, and GaAsSb, with controllable thickness and morphology on arbitrary substrates. Utilizing a low-cost, wafer-scale vapor deposition process compatible with standard semiconductor procedures, these Ga-based films can be patterned for various functional applications. For example, the patterned Ga-based thin films exhibit broad applicability in p-channel transistor arrays (with hole mobility of 0.25 cm^2^ V⁻^1^ s⁻^1^), functional synaptic devices, and flexible omnidirectional imaging sensors (maintaining functionality at incident angles as low as 5°). Overall, the proposed induced fit growth method facilitates the growth of Ga-based semiconductor films with greater integration flexibility, enhancing their advanced functionality and broad applicability.

## Abstract (web summary)

Inspired by the induced fit theory in molecular biology, this work successfully prepared Ga-based semiconductor films with weak substrate dependence, holding great promise for next-generation electronics and optoelectronics.

## Graphical abstract

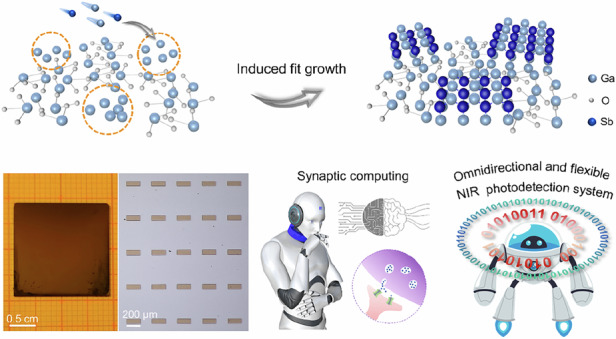

## Introduction

Current fabrication methods for semiconductor thin films, which typically rely on post-synthesis assembly and epitaxial growth, face challenges related to processing complexity and high growth temperatures^1–8^. These methods are not fully optimized for the demands of modern Internet of Things (IoT) applications^5,6^. Consequently, existing semiconductor thin films and their production techniques fail to achieve efficient optoelectronic conversion, robust flexibility, transparency, and synaptic behavior required for future multifunctional applications^7–11^.

At the same time, among many semiconductor materials, Ga-based thin films, such as Ga_2_O_3_, GaN, GaSe, GaAs, GaSb, and GaAsSb, are known for their high carrier mobilities, tunable bandgaps ranging from ultraviolet to infrared, efficient optical absorption and emission, and adjustable defect and carrier concentrations. These properties are essential in high-speed electronics, broadband photodetection, multicolor light-emitting diodes, and neuromorphic computing^12–19^. Furthermore, functional substrates like flexible mica, transparent glass, ferroelectric oxides, high thermal conductivity substrates, and superconductor substrates enhance the potential of these semiconductor films in wearable optoelectronics, three-dimensional (3D) optical imaging systems, nonvolatile memories, radiofrequency electronics, and spintronics^7,11,20–22^. Therefore, developing feasible growth methods for Ga-based semiconductor films on various functional substrates is crucial to facilitate their multifunctionality and scalability.

In general, substrate lattice matching or buffer layers are required to grow semiconductor films^22–26^. However, inspired by induced fit theory in molecular biology, where a substrate induces a conformational change in an enzyme to facilitate binding, we propose an analogous approach for the universal growth of Ga-based semiconductor films on functional substrates^27,28^. Zavabeti et al. demonstrated that an amorphous GaO_x_ film with residual free Ga atoms could be exfoliated from a liquid Ga surface using a squeeze-printing method^29^. The residual free Ga atoms in the exfoliated amorphous GaO_x_ film can be considered analogous to the specific enzyme in induced fit theory, promoting the growth of Ga-based semiconductor films.

In this work, we present a novel induced fit growth method for wafer-scale multifunctional Ga-based semiconductor films, such as GaSb, GaSe, GaAs, and GaAsSb, on functional substrates including Si/SiO_2_, transparent glass, and flexible mica. The thickness and surface roughness of the as-prepared Ga-based semiconductor films can be precisely controlled by adjusting the growth temperature and duration. Furthermore, these films can be patterned into desired shapes, enhancing their utility in flexible electronics and omnidirectional photodetection applications. For instance, GaSb films exhibit typical p-type conductive behavior with a high current density of 0.1 μA μm^-1^ and high hole mobility of 0.25 cm^2^ V^-1^ s^-1^, along with large-scale uniformity. Enabled by the abundant traps in the interfacial GaO_x_ film, the patterned GaSb film transistors demonstrate impressive synaptic behaviors. When deposited on transparent glass and flexible mica, the resulting omnidirectional and flexible photodetectors maintain a good photoresponse even at an incident angle of 5° and after 900 folding cycles. These findings highlight a successful strategy for the induced fit growth of multifunctional Ga-based semiconductor films on arbitrary substrates, paving the way for advancements in flexible and multifunctional electronic devices.

## Results

### Induced fit growth of Ga-based semiconductor thin films

**Fig. 1:**
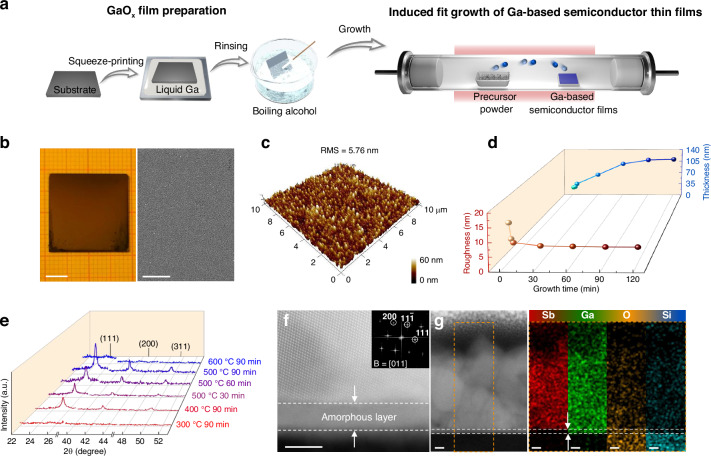
Induced fit growth of Ga-based semiconductor thin films. **a** Schematic diagrams of the induced fit growth of Ga-based semiconductor films. **b,** **c** Optical photograph, SEM, and 3D AFM images of the as-prepared GaSb film. The scale bars are 0.5 cm and 5 μm, respectively. **d** Roughness and thickness of the GaSb films prepared with different growth durations. **e** XRD patterns of the GaSb films prepared at different growth temperatures and durations. **f** Cross-sectional HRTEM image of the GaSb film. The inset shows the corresponding FFT pattern. The scale bar is 5 nm. **g**, STEM image and EDS elemental mapping images of Sb, Ga, O, and Si. The scale bars are 10 nm

Here, the GaSb thin films are prepared using an induced fit growth method, while the experimental details are shown in the “Materials and Methods” section. In short, as shown in Fig. 1a, the GaO_x_ film is prepared first on arbitrary substrates by a reported liquid metal van der Waals exfoliation method^30,31^. As verified by X-ray diffraction (XRD) in Fig. S1, no diffraction peak is observed for the as-exfoliated GaO_x_ film, demonstrating its amorphous properties. The compact surface and uniform morphology of the as-exfoliated GaO_x_ thin film are further verified by the atomic force microscope (AFM), as shown in Fig. S2. Then, the as-exfoliated amorphous GaO_x_ film is utilized to induce fit growth of Ga-based semiconductor thin films in a furnace. Figure 1b, c are the optical photograph, scanning electron microscope (SEM), and 3D AFM images of the as-prepared GaSb film, showing good uniformity of 1.8 cm × 1.8 cm, compact surface morphology, and root mean square (RMS) roughness of 5.76 nm. In fact, the thickness, surface roughness, and crystallinity of GaSb films can be controlled well by growth durations and temperatures. As shown in Fig. 1d, the thickness increases from 22 nm to 108 nm, and the RMS roughness decreases from 14.1 nm to 5.76 nm with the prolongation of growth duration. These values remain almost unchanged after 90 min. The thickness and RMS roughness are deduced from AFM images of Fig. S3. Accordingly, the crystallinity of GaSb films is checked by XRD in Fig. 1e, inferring the as-prepared films to have the zinc blende crystal structure (JCPDS Card No. 07-0215)^32,33^. By optimizing growth temperatures from 300 to 600 °C, more substantial diffraction peaks are observed at 500 °C. No diffraction peaks of GaSb are observed for the growth temperatures of 300 and 600 °C. Meanwhile, the diffraction peaks become stronger when the growth duration extends to 90 min. Scanning transmission electron microscopy (STEM) and energy-dispersive X-ray spectroscopy (EDS) are further adopted to study the crystallinity and stoichiometry of as-prepared GaSb films, as shown in Fig. 1f, g and Fig. S4. As depicted in Fig. 1f, continuous lattice fringes are observed in the cross-sectional GaSb film, and clear reciprocal lattice spots are extracted by fast Fourier transform (FFT), indicating the good crystallinity characteristic. Notably, an obvious amorphous layer with a thickness of 0.37 nm exists between GaSb and the Si/SiO_2_ substrate. The thickness is in line with the result of AFM in Fig. S2. As displayed in Fig. 1g, the composition of the amorphous layer is Ga and O, and the film is Ga and Sb, illustrating that an amorphous GaO_x_ film is located between the GaSb film and the Si/SiO_2_ substrate. Owing to the excellent thermodynamic and chemical stability, the amorphous GaO_x_ thin film’s thickness remains constant during the GaSb thin film’s growth process^34,35^. In a word, GaSb films with controlled thickness, surface roughness, and crystallinity can be successfully prepared by the induced fit growth method.

### Growth mechanism of GaSb films by the induced fit growth method

**Fig. 2:**
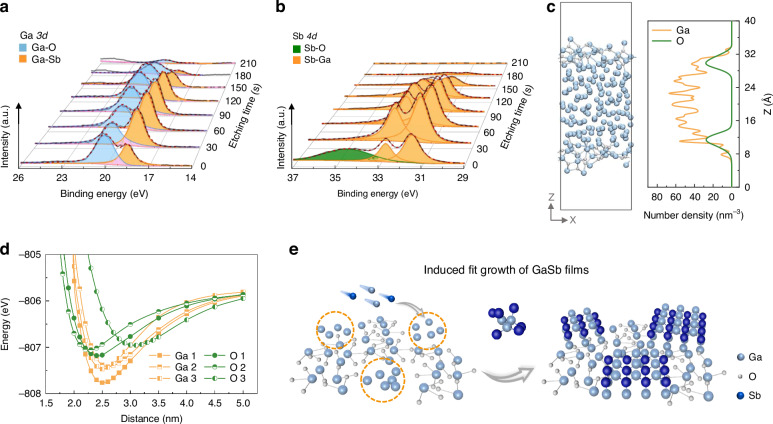
Growth mechanism of the induced fit growth method. **a,** **b** XPS spectra of Ga *3* *d* and Sb *4* *d* of the GaSb films versus etching times. **c** Schematic diagram and the number density distribution of Ga and O atoms along the Z-axis in the optimized GaO_x_ unit cell. **d** Energy variation curves of the introduced Sb atom as it gradually approaches three selected Ga or O atoms. **e** Schematic diagram of the induced fit growth method for GaSb films

For guiding the growth of multifunctional semiconductor thin films on arbitrary substrates, X-ray photoelectron spectroscopy (XPS) and first-principles calculations are utilized to study the induced fit growth mechanism of GaSb films, as depicted in Fig. 2 and Figs. S5-8. All XPS peaks are calibrated with the C *1* *s* peak (284.8 eV) and fitted through Gauss-Lorentz fitting. From the XPS spectra of Ga *3* *d* and Sb *4* *d* in Fig. S5, the primary/shoulder peaks centered at 20.0/17.9 eV are observed for the as-exfoliated GaO_x_ film, corresponding to the Ga-O bond and metal Ga, respectively^18^. This indicates that some Ga atoms are completely free in the as-exfoliated GaO_x_ film^31,36,37^. This result is similar to the film prepared at a low growth temperature of 300 °C. When the growth temperature reaches 400 °C, peaks of the Ga-Sb bond (at 18.5 eV in Ga *3* *d* and 31.3/32.6 eV in Sb *4* *d*) are observed. However, when the growth temperature reaches 600 °C, only peaks of the Ga-O bond are observed. At the same time, the film prepared at 500 °C shows more substantial Ga-Sb peaks than that prepared at 400 °C. All these results demonstrate the optimal growth temperature of GaSb film as 500 °C, which is in line with the results of XRD in Fig. 1e.

XPS with Ar^+^ ion etching is further performed to shed light on the growth mechanism, particularly focusing on the interface between the as-exfoliated GaO_x_ and the as-prepared GaSb film. As shown in Fig. 2a, b, with the prolongation of etching time, the peak intensity of the Ga-O bond first decreases to a constant value and then further reduces to a minimum value. At the same time, the peak intensity of the Ga-Sb bond initially increases, then gradually decreases until it disappears. The peak of the Sb-O bond is only observed in the as-prepared GaSb film. These findings demonstrate that a native oxide layer on the GaSb film surface and a GaO_x_ film exist at the interface of the GaSb film and the growth substrate. Notably, no peak of metal Ga is observed in Fig. 2a, indicating that it is involved in the growth of GaSb film. To further verify the proposal, the spin-coating GaO_x_ film is also prepared for the growth of GaSb film, as shown in Figs. S6-S7. As shown in the AFM image, the surface of the spin-coated GaO_x_ thin film is similar to that of the as-exfoliated GaO_x_ thin film. However, compared with the as-exfoliated GaO_x_ film, no peaks of free metal Ga are observed in the XPS of the spin-coating GaO_x_ film. Using this spin-coating GaO_x_ film, GaSb film cannot grow at any growth temperature. Obviously, it can be inferred that the free metal Ga in the as-exfoliated GaO_x_ film facilitates the growth of the GaSb film.

To gain deeper insight into the growth mechanism, simulation computing based on first-principles calculations is performed in Fig. 2c, d. As shown in Fig. 2c, the Ga atoms tend to diffuse outward over time, and the O atoms tend to penetrate from the surface into the body, which results in an as-exfoliated Ga-rich GaO_x_ film. This phenomenon is further verified by the atomic number density profile along the z-axis. Due to free diffusion, some Ga atoms relocate to positions outside the O atoms. Furthermore, as depicted in Fig. 2d and Fig. S8, to explore the thermodynamic behaviors of Sb vapor atoms during the induced fit growth process, the simulations are conducted in which a single Sb atom approaches three randomly selected O atoms or Ga atoms on the surface of the GaO_x_ film, respectively. The binding energy between Sb and Ga atoms is always lower than that between Sb and O atoms, proving that the introduced Sb atoms bind to Ga atoms more easily than O atoms. The results above reveal that the Ga-rich GaO_x_ film facilitates the deposition of Sb vapor atoms, thereby promoting the growth of uniform and compact GaSb films. In this case, the schematic diagram of the induced fit growth method for GaSb films is shown in Fig. 2e. The free metal Ga in the as-exfoliated GaO_x_ film promotes the deposition of Sb vapor, subsequently leading to the island growth of the GaSb film. With the prolonged growth duration, the film gradually becomes compact and uniform. In brief, the as-proposed induced fit growth method paves the way for the growth of multifunctional semiconductor thin films on arbitrary substrates.

### Patterning and electrical properties of GaSb thin films

**Fig. 3:**
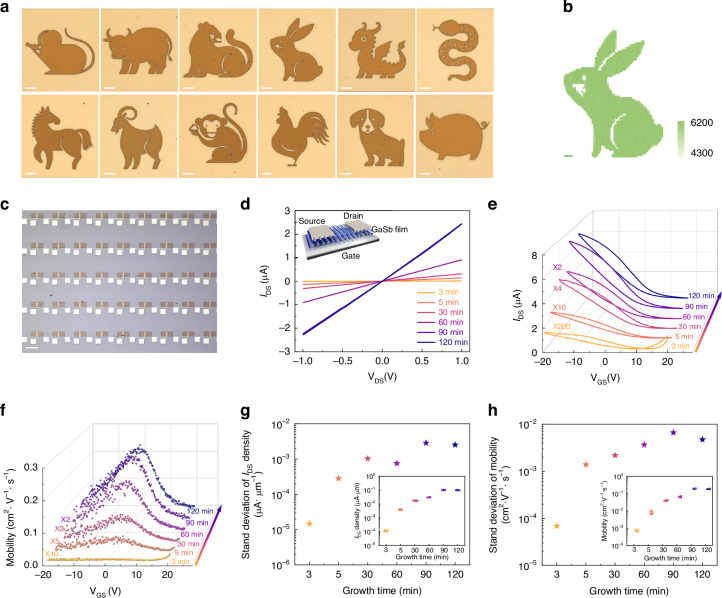
Patterning and electrical properties of the as-prepared GaSb thin films. **a** Optical images of the Chinese zodiac patterned from the as-prepared GaSb thin films. All scale bars are 50 μm. **b** Raman mapping of a patterned rabbit. The scale bar is 20 μm. **c** Optical image of a patterned transistor array. The scale bar is 200 μm. **d–f** Output, transfer, and mobility characteristics of the as-patterned GaSb films transistors. **g**, **h** Standard variations of I_ds_ density and mobility versus growth duration. The insets are data collected from 20 devices

Microscale patterning is essential but still challenging when using semiconductor thin films for practical applications^38,39^. As shown in Fig. 3, all the induced fit-grown GaSb films can be patterned, and the patterning details can be found in the “Materials and Methods” section. As displayed in the optical images of Fig. 3a, the Chinese zodiac is patterned from the as-prepared GaSb films, exhibiting micrometer-level resolution. The large-scale uniformity is verified by Raman mapping, as presented in Fig. 3b and Fig. S9. With the good patterning ability, the as-prepared GaSb film is then constructed successfully as a transistor array, as illustrated in Fig. 3c, for studying electronic and optoelectronic behaviors. Figure 3d, e show the representative output and transfer characteristics of the as-constructed GaSb thin film transistors, respectively, confirming the ohmic-like contacts between the GaSb films and the Ni electrodes. The output curves versus different V_GS_ for the 90-min-grown GaSb thin film transistor are exhibited in Fig. S10. With the V_GS_ increasing from -20 V to 20 V, the I_DS_ decreases linearly from 7.1 μA to 1.0 μA at the V_DS_ of 1.0 V. The p-type conducting behavior of the as-prepared GaSb films is exhibited. The I_DS_ increases from 0.75 × 10^-2^ to 7.2 μA for the prolonged growth duration. Notably, as depicted in Fig. S11, all the leakage currents are about 100-300 pA, significantly lower than the I_DS_. As displayed in Fig. 3f, the peak hole mobility also increases with the growth duration, reaching a maximum value of 0.25 cm^2^·V^-1^·s^-1^ at a duration of 90 min. The standard variations of I_ds_ density and mobility are then collected from 20 devices and presented in Fig. 3g, h, further verifying the device performance uniformity of the as-constructed GaSb transistor array. When the growth duration increases from 3 min to 120 min, the standard variation of I_DS_ density increases from 1.5 × 10^-5^ to 2.5 × 10^-3^ μA μm^-1^, along with the standard variation of peak hole mobility increasing from 6.8×10^-5^ to 4.7 × 10^-3 ^cm^2^ V^-1^ s^-1^. The minor standard deviations of all transistor arrays further verify the uniformity of the as-prepared GaSb films. In short, the induced fit-grown p-type GaSb films are patternable, illustrating their potential as active materials for electronic and optoelectronic devices.

### Synaptic behaviors of GaSb thin films

**Fig. 4:**
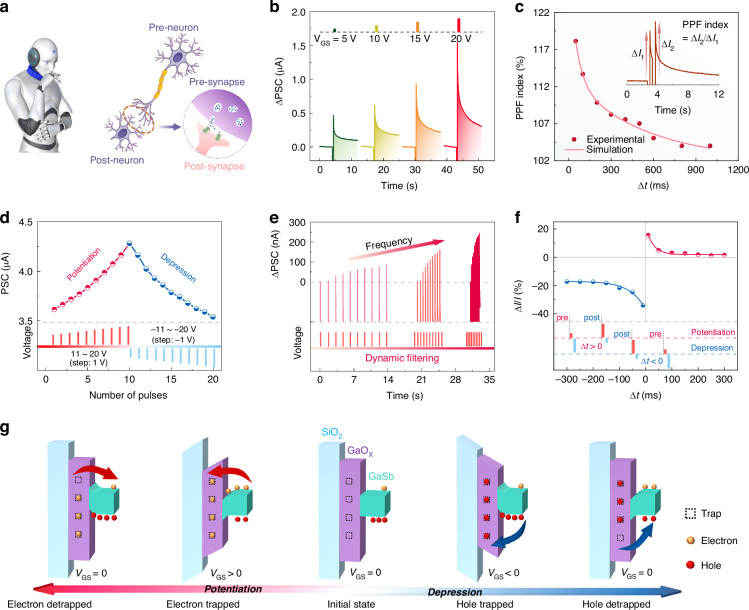
Synaptic behaviors of the as-prepared GaSb thin films. **a** Schematic diagram of machine artificial intelligence and biological synapse. **b** Synaptic plasticity triggered by positive V_GS_ pulses. **c** PPF index as a function of V_GS_ pulse interval time (V_GS_ = 10 V, width = 100 ms), and the inset is the PSC triggered by a pair of V_GS_ pulses. **d** LTP and LTD realized by continuously applying 20 V_GS_ pulses (width = 100 ms, interval time = 100 ms). **e** SRDP behavior versus different pulse frequencies (V_GS_ = 10 V, width = 100 ms). **f** STDP behavior versus different Δt (V_GS_ = 5/-20 V for pre-stimulation and V_GS_ = 20/-5 V for post-stimulation, width = 100 ms). **g** Proposed charge-trapping model of the as-constructed GaSb film synaptic transistor. All the measurements are under a bias of 1 V

Realizing that artificial machine intelligence is important for developing neuromorphic computing and the Internet of Things. The synaptic behaviors of the as-constructed 90-minute-grown GaSb thin film transistors are studied in Fig. 4. Figure 4a shows the machine artificial intelligence and biological synapse schematic. As illustrated in Fig. 4b, a postsynaptic current (PSC) can be triggered by a V_GS_ pulse acting on the GaSb film transistor, and the change in PSC is expressed as ΔPSC. PSC decreases when +V_GS_ pulses (width = 100 ms) are applied and quickly reaches peak values when +V_GS_ pulses are removed, then attenuates slowly. These results closely resemble the excitatory PSC (EPSC) process in biological synapses^40,41^. The V_GS_ pulses increase from 5 to 20 V, and the ΔPSC increases from 0.47 to 1.5 μA, inferring the successful transition of short-term plasticity (STP) to long-term plasticity (LTP). The inverse synaptic behavior is observed in Fig. S12. When -V_GS_ pulses are applied and removed, exhibiting programmable depression behaviors. Moreover, as shown in Fig. S13, all the as-prepared GaSb thin film synaptic transistors exhibit V_GS_-dependent synaptic behaviors with uniform responses to identical external V_GS_ stimulations, verifying the robust reliability and uniformity. As a typical biological synaptic behavior, paired pulse facilitation (PPF), a phenomenon where synaptic weight stimulated by the second spike increases when it closely follows the first, plays a vital role in decoding temporal information in auditory or visual signals. As depicted in the inset of Fig. 4c, the PSC triggered by the second V_GS_ pulse (ΔI_2_) is larger than that triggered by the first pulse (ΔI_1_), a typical PPF behavior characteristic^41^. The PPF is described using the following equation: PPF = 1 + C_1_exp(Δt/τ_1_) + C_2_exp(Δt/τ_2_), in which C_1_ and C_2_ are the initial facilitation magnitudes of rapid and slow phases, τ_1_ and τ_2_ are the characteristic relaxation times of the rapid and slow decay, respectively, and Δt is the pulse interval. The values of τ_1_ (53.5 ms) and τ_2_ (541.3 ms) are exhibited. The PPF index, defined as ΔI_2_/ΔI_1_, can reach up to 118% when Δt is 50 ms. It decreases exponentially as the Δt increases. The LTP and long-term depression (LTD) are also successfully mimicked via a series of electrical stimulations in Fig. 4d, exhibiting good linearity and symmetry. Next, the Hebbian learning rule is mimicked by the GaSb thin film transistor, including spike rate-dependent plasticity (SRDP) and spike timing-dependent plasticity (STDP). As shown in Fig. 4e, ΔPSC increases with the V_GS_ frequency, and the dynamic filtering is realized, demonstrating SRDT behavior. On the other hand, STDP characterizes the synaptic weight change resulting from the temporal interval. As depicted in Fig. 4f, the arrival orders of pre- and post-stimulations determine potentiation or depression, indicating that the synapse shows potentiation/depression when Δt is positive/negative. In a word, various typical synaptic behaviors, including STP, LTP, PPF, SRDP, and STDP, are successfully mimicked by the GaSb thin film transistors.

Notably, to validate the mechanism that enables synaptic behaviors, the top-gated GaSb thin film transistor is constructed and studied in Fig. S14. Compared with the back-gated transistor, there is no amorphous GaO_x_ film between the dielectric and the GaSb film in the as-constructed top-gated transistor. When the positive or negative V_GS_ pulses are applied, no obvious synaptic behaviors are observed. Based on the above results, a charge-trapping model is proposed to explain the synaptic behaviors of the as-prepared GaSb thin film transistor, in which the amorphous GaO_x_ film serves as the charge trap layer to capture carriers, as depicted in Fig. 4g. The middle picture represents the initial state. When a +V_GS_ (-V_GS_) pulse is applied to the back gate, electrons (holes) are driven to the amorphous GaO_x_ film and trapped there. Once the +V_GS_ (-V_GS_) pulse is removed, the trapped electrons (holes) turn the GaSb channel to an on-state (off-state), representing the potentiation (depression). As time passes, the trapped electrons (holes) would be released, leading to decreased (increased) hole carriers in the GaSb channel. Therefore, benefiting from the use of amorphous GaO_x_ film during the induced fit growth process, the as-prepared GaSb thin films are promising for developing neuromorphic computing.

### Photodetection behaviors of GaSb thin films

**Fig. 5:**
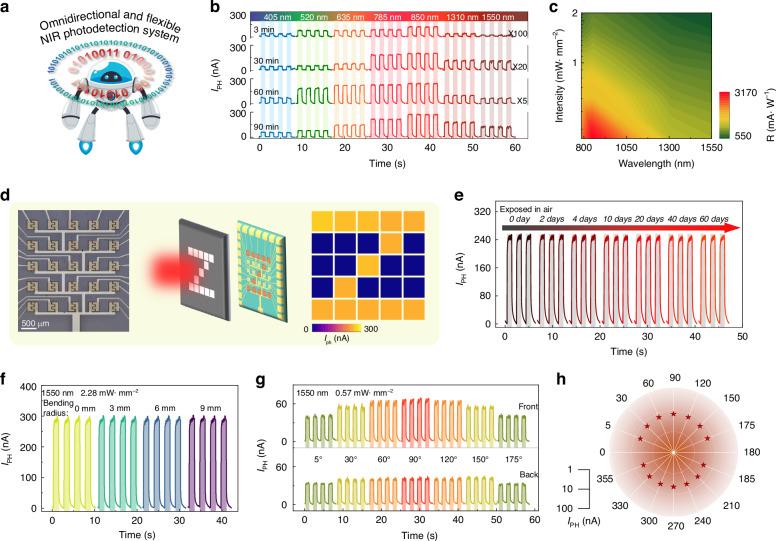
Photodetection behaviors of the as-prepared GaSb thin films. **a** Schematic diagram of the omnidirectional and flexible NIR photodetection system. **b** Wavelength-dependent broadband photodetection behaviors of the as-prepared GaSb films. The laser intensity is 0.57 mW·mm^-2^. **c** Two-dimensional contour map of R versus different wavelengths and laser intensities. **d** Demonstration of the NIR photodetection imaging system. **e** Air stability of the as-studied NIR photodetector. **f** Time-resolved photoresponse of the as-fabricated flexible NIR photodetector after different bending radii. **g**, **h** Omnidirectional photodetection behaviors of the as-fabricated NIR photodetector. All the measurements are under a bias of 1 V

Flexibility and transparency are critical requirements for the omnidirectional and flexible NIR photodetection system, as shown in Fig. 5a. In this regard, the photodetection performance of the as-fabricated GaSb thin film transistors is investigated and presented in Fig. 5b–e. As expected, all the as-fabricated photodetectors exhibit broadband photodetection behaviors, owing to the narrow bandgap of GaSb (0.72 eV), as depicted in Fig. 5b and Figs. S15-S16. With the increasing thin film growth duration, the photocurrent (I_PH_ = I_light_ − I_dark_) increases from 8 to 274 nA under the illumination of an 850 nm laser with an intensity of 0.57 mW·mm^-2^. This phenomenon may result from the increased thickness of the thin films. At the same time, the I_PH_ can be effectively modulated by periodic laser illumination, demonstrating reproducible dynamic stability. Another two critical parameters of photodetectors, responsivity (R) and detectivity (D*) of the GaSb film grown by 90 min, are studied in Fig. 5c and Fig. S17. Generally speaking, R can be defined as R = I_PH_/(PA), and D* can be defined as D* = RA^1/2^/(2eI_dark_)^1/2^, in which P is the incident power density, A is the effective irradiated area, e is the electronic charge, and I_dark_ is dark current^42,43^. As a result, the maximum R and D* can be as high as 3170 mA·W^-1^ and 5.3 × 10^9^ Jones under the illumination of an 850 nm laser with an intensity of 0.28 mW mm^-2^. As shown in Fig. S18, the response time, including rise time (τ_r_) and decay time (τ_d_), is defined as the time for the photocurrent to increase/decrease from 10/90% to 90/10%^44,45^. Obviously, the as-constructed photodetector exhibits τ_r_ of 11 ms and τ_d_ of 18 ms, respectively, comparable with the photodetectors based on other semiconductor thin films^46,47^. Because of the excellent NIR photodetector performance, a 5 × 5-pixel NIR imaging array is constructed by the as-prepared GaSb thin film, as illustrated in Fig. 5d. A hollow target mask of the letter “Z” is placed before the photodetector array. The precise and uniform imaging results demonstrate the excellent NIR photoresponse and uniformity of the as-prepared GaSb thin film. Then, the air stability of the as-prepared GaSb thin film photodetector is also evaluated and compiled in Fig. 5e. No apparent attenuation of I_PH_ is observed after exposure to ambient for 60 days, designating excellent air stability and robust operation of the as-prepared GaSb thin film photodetector.

Benefiting from the weak substrate dependence of the induced fit growth method, GaSb thin films can be readily prepared on various functional substrates, such as flexible mica and rigid glass. Correspondingly, flexible and omnidirectional NIR photodetectors can be fabricated and examined. All fabricated photodetectors exhibit ohmic contact. By adopting the flexible and transparent growth substrate of mica, the photoresponse of the as-prepared GaSb thin film maintains even after 900 bending-recovering cycles and bending at a radius of 3 mm under the illumination of a 1550 nm laser, exhibiting good bendability and flexibility, as shown in Fig. 5f and Fig. S19. When the laser irradiates the photodetector in omnidirectional directions, the variation of I_PH_ is recorded and shown in Fig. 5g, h. Notably, a stable photoresponse can be observed at an incident angle of 5°. The I_PH_ can keep over 64% compared to that at an incident angle of 90°, indicating excellent omnidirectional photodetection performance. This excellent omnidirectional photodetection performance can also be observed when rigid glass is used as the growth substrate, as shown in Fig. S20. As a result, due to the induced fit growth process, the as-prepared GaSb thin films are promising as active channels for omnidirectional and flexible NIR photodetection systems for wearable devices.

### Versatility of the induced fit growth method

**Fig. 6:**
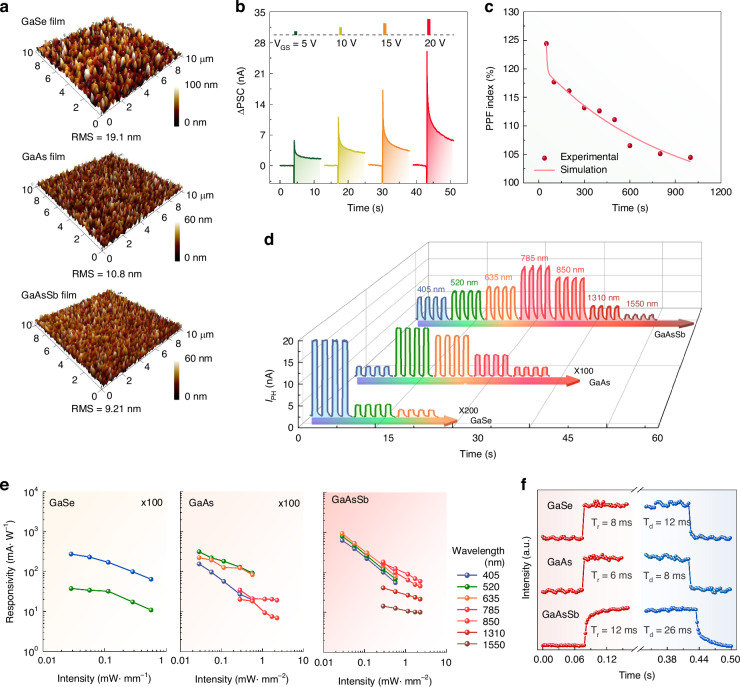
Versatility of the induced fit growth method for multifunctional Ga-based semiconductor thin films. **a** 3D AFM images of the as-prepared GaSe, GaAs, and GaAsSb thin films. **b,** **c** Synaptic plasticity and PPF behaviors of the GaAsSb thin film transistor. **d** Wavelength-dependent broadband photodetection behaviors of the as-prepared GaSe, GaAs, and GaAsSb thin films. The laser intensity is 0.57 mW·mm^-2^. **e,** **f** Wavelength-dependent R and temporal photoresponse of GaSe, GaAs, and GaAsSb thin films. All the measurements are under a bias of 1 V

Owing to the free metal Ga on the as-exfoliated GaO_x_ film, the induced fit growth method can also be demonstrated as a successful growth approach for other Ga-based semiconductor thin films, such as GaSe, GaAs, and GaAsSb, as shown in Fig. 6. Figure 6a and Fig. S21 illustrate the AFM images of the films, and the thicknesses of the GaSe, GaAs, and GaAsSb films are 204 nm, 92 nm, and 173 nm, along with the RMS roughnesses of 19.1 nm, 10.8 nm, and 9.21 nm, respectively. All these films display a compact surface morphology. The XRD patterns and Raman spectra in Fig. S22 confirm their good crystallinity and uniform composition. As shown in Fig. S23, the as-prepared GaAsSb, GaAs, and GaSe thin films exhibit p-type, n-type, and p-type conducting behaviors, respectively. Similar to the GaSb counterpart, the GaAsSb thin film transistor also displays fantastic synaptic behaviors in Fig. 6b, c and Fig. S24 due to the charge-trapping role of GaO_x_ film at the interface of GaAsSb film and growth substrate. As the V_GS_ pulses increase from 5 to 20 V, the successful transition of STP to LTP is observed in Fig. 6b. As shown in Fig. 6c, the PPF index of the GaAsSb film synaptic transistor can reach up to 125% when Δt is 50 ms. Furthermore, the photodetection behaviors of the as-prepared Ga-based semiconductors are also studied in Fig. 6d–f and Fig. S25. With the bandgaps covered from UV to NIR, all these Ga-based semiconductors show excellent broadband photoresponse. The I_PH_ can be effectively modulated by periodic laser illumination, demonstrating reproducible dynamic stability. The maximal R values of GaSe, GaAs, and GaAsSb films are 940 mA·W^-1^, along with the maximal D* values of 5.7 × 10^9^ Jones. At the same time, the corresponding τ_r_/τ_d_ values of GaSe, GaAs, and GaAsSb films are 8/12, 6/8, and 12/26 ms, respectively. The induced fit growth method paves an efficient way to the controllable growth of multifunctional Ga-based semiconductor thin films.

## Discussion

Recently, many growth technologies, such as interfacial epitaxy methodology, surface chemistry of growth substrates, and modularized growth strategy, have been developed for the wafer-scale growth of Van der Waals semiconductors, such as the transition-metal dichalcogenides^48–53^. In this work, we have successfully prepared large-scale multifunctional Ga-based semiconductor thin films, including GaSb, GaSe, GaAs, and GaAsSb, on various substrates using an induced fit growth method. The thickness and surface roughness of these films can be effectively controlled by adjusting the growth temperature and duration. Notably, the GaSb films exhibit easy patterning capabilities and have been utilized to construct transistors, which display typical p-type conductive behaviors and synaptic functions. Additionally, when GaSb films are prepared on transparent glass and flexible mica, the resulting omnidirectional and flexible photodetectors maintain excellent photoresponse even at an incident angle of 5° and after 900 folding cycles. These results highlight the efficacy of the induced fit growth strategy for Ga-based semiconductor thin films. Furthermore, the growth and photoelectronic properties of other Ga-based semiconductor films, such as GaSe, GaAs, and GaAsSb, have also been successfully demonstrated. These findings underscore a straightforward and effective strategy for the induced fit growth of multifunctional semiconductor thin films.

It is worth pointing out that GaSb has been considered a promising thermoelectric material, owing to its high Seebeck coefficient and good electrical conductivity^54^. However, the relatively high thermal conductivity limits its thermoelectric figure of merit, ZT^55^. To improve the thermoelectric performance, further works can focus on doping or nanostructuring large-scale GaSb thin films using this induced fit growth method^55,56^. With high optical absorption and a direct bandgap, GaAs provides advantages in solar cells^57^. Moreover, GaAs is resistant to heat and radiation damage due to the higher threshold energy under high-energy radiation, promising for radiation-resistant devices^58^. In the case of GaSe, it shows room-temperature ferroelectricity, benefiting from the unique intralayer sliding and atomic vacancy defect, which promises nonvolatile memory and optoelectronic devices^59,60^. In short, the as-studied Ga-based semiconductor thin films grown by the induced fit growth method also significantly promise the future high-performance thermoelectric devices, solar cells, ferroelectric memory, etc.

## Materials and Methods

### Induced fit growth of Ga-based semiconductor films

Ga-based semiconductor films are prepared by a proposed induced fit growth method in a dual-zone horizontal tube furnace. The liquid metal van der Waals exfoliation method is used to prepare the GaO_x_ film. The metal Ga remains liquid when placed on glass under heating. The substrate is gently squeezed vertically onto the surface of the droplet. When the substrate is lifted, the continuous and uniform GaO_x_ film is transferred onto the surface of the substrate. The visible residual liquid Ga droplets are removed by rinsing with boiling alcohol. Next, the high-purity Ga-based semiconductor powders (99.999% purity) are placed in the upstream zone, while the growth substrates with GaO_x_ films are placed in the downstream zone. The upstream zone is heated to 750, 850, 800, and 770 °C, while the downstream zone is heated to 500, 400, 550, and 500 °C for the growth of GaSb, GaSe, GaAs, and GaAsSb films, respectively. The heating rate is set to 10 °C/min. The system is pumped to 7×10^-3 ^Torr. Upstream precursor steam is transported by nitrogen (99.999% purity, 50 sccm) to the downstream zone. When it reaches the set time, the upstream and downstream zones stop together and cool to room temperature under the nitrogen flow.

### Material characterizations

The morphologies are characterized using a microscope (Olympus microscope BX53M) and SEM (Helios G4 UC, Thermo Scientific). The morphologies and heights of the as-prepared films are characterized by AFM (Dimension Icon). The crystal phase and phase purity of the as-prepared films are verified by XRD (D8 Advance, Bruker). The microstructure and composition characterizations are characterized by TEM and HRTEM associated with EDS mapping (Spectra 300, Thermofisher). The chemical composition and elemental valence are analyzed by XPS (Nexsa, Thermo Scientific). Raman (Nanobase XperRAM) spectra and mappings are obtained with 532 nm excitation wavelengths.

### Film etching

The negative photoresist or Polymethyl Methacrylate (PMMA) is spin-coated on the sample surface, and then standard photolithography or electron beam lithography is adopted. After the developing process, a tetramethylammonium hybrid solution is used for film etching. Then, 1-Methyl-2-pyrrolidinone or acetone removes the residue photoresist or PMMA.

### Device fabrication and measurements

A copper grid is placed on the top of the as-prepared films as a shadow mask for resist-free metal evaporation. A 50 nm thick Ni film is deposited as contact metal via e-beam evaporation. A SourceMeter (Keithley 2602B) is used to assess the electrical performance in response to applied electrical pulses. In an atmospheric environment, electrical and photodetection performance is characterized by a standard electrical probe station and a semiconductor analyzer (Agilent B1500A). A semiconductor analyzer is the current signal acquisition equipment, and diode lasers are the light source. The laser intensity is calibrated by a power meter.

### Simulation calculation

The first-principles calculations are carried out using the density functional theory in the Vienna Ab initio Simulation Package using the plane-wave pseudopotential method^61^. The generalized gradient approximation in the form of Perdew–Burke–Ernzerhof is used to describe the exchange-correlation of electrons^62^. The energy cutoff of the plane wave is set as 500 eV, and the energy and force convergence thresholds are set to be 10^−6^ eV and 10^−2^ eV/Å, respectively. The Grimme method (DFT-D3) is used to correct the weak Van der Waals interaction^63^. The GaO_x_ unit cell is obtained by expanding the Ga elemental cell and substituting the Ga atoms in both the top and bottom two atomic layers with O atoms in a 1:1 ratio. The molecular dynamics simulations are performed for 50,000 steps with a 1 fs time step. A Nosé thermostat maintains the system temperature at 350 K throughout the simulation^64^. The Brillouin zone is sampled with a k-point mesh of 1 × 1 × 1 for the rectangular unit cell of GaO_x_.

## Supplementary information

Supplementary Information

## Data Availability

All data that support the findings of this work are available within the paper. Additional data are available from the corresponding authors upon request.
