# Supplementary material for: Induced fit growth of Ga-based semiconductor thin films for brain-inspired electronics and optoelectronics
Source: Light Sci Appl. 2026 Feb 4;15:103. doi: 10.1038/s41377-025-02096-2 (PMC12867999; doi:10.1038/s41377-025-02096-2)
Supplement: Supplementary file 1 — Supplementary Information [file 41377_2025_2096_MOESM1_ESM.docx]

Supplementary Information for

**Induced fit growth of Ga-based semiconductor thin films for brain-inspired electronics and optoelectronics**

Zixu Sa^1,#^, Kepeng Song^2,#^, You Meng^3,#^, Wenfeng Wu,^1,#^ Zhaocong Wang,^1^ Pengsheng Li,^1^ Jie Zhang,^1^ Zeqi Zang,^1^ Guangcan Wang,^1^ Mingxu Wang,^1^ Zhitai Jia,^1^Yang Tan,^1^ Weifeng Li,^1,*^ SenPo Yip,^4^ Feng Chen^1,*^, Johnny C. Ho^3,4,5,*^, and Zai-xing Yang^1,*^

^1^School of Physics, State Key Laboratory of Crystal Materials, Shandong University, Jinan, 250100, China

^2^School of Chemistry and Chemical Engineering, Shandong University, Jinan, 250100, China

^3^Department of Materials Science and Engineering, City University of Hong Kong, Hong Kong, 999077, China

^4^Institute for Materials Chemistry and Engineering, Kyushu University, Fukuoka, 816-8580, Japan

^5^State Key Laboratory of Terahertz and Millimeter Waves, City University of Hong Kong, Hong Kong, 999077, China

Corresponding Author:

*Address Correspondence to W.F. Li (lwf@sdu.edu.cn), F. Chen ([drfchen@sdu.edu.cn](mailto:drfchen@sdu.edu.cn)), J.C. Ho (johnnyho@cityu.edu.hk), and Z.-x. Yang (zaixyang@sdu.edu.cn).


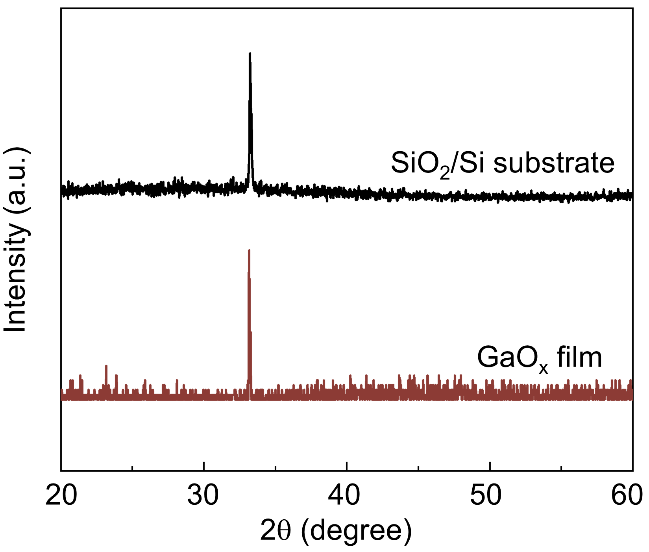


**Fig. S1** XRD patterns of the substrate and GaO_x_ thin film.

Fig. S1 shows the typical XRD patterns of the substrate and GaO_x_ thin film. The amorphous characteristic of GaO_x_ thin film is demonstrated. The distinct peaks centered at 33° are attributed to (200)-orientation of Si substrates.^1-3^


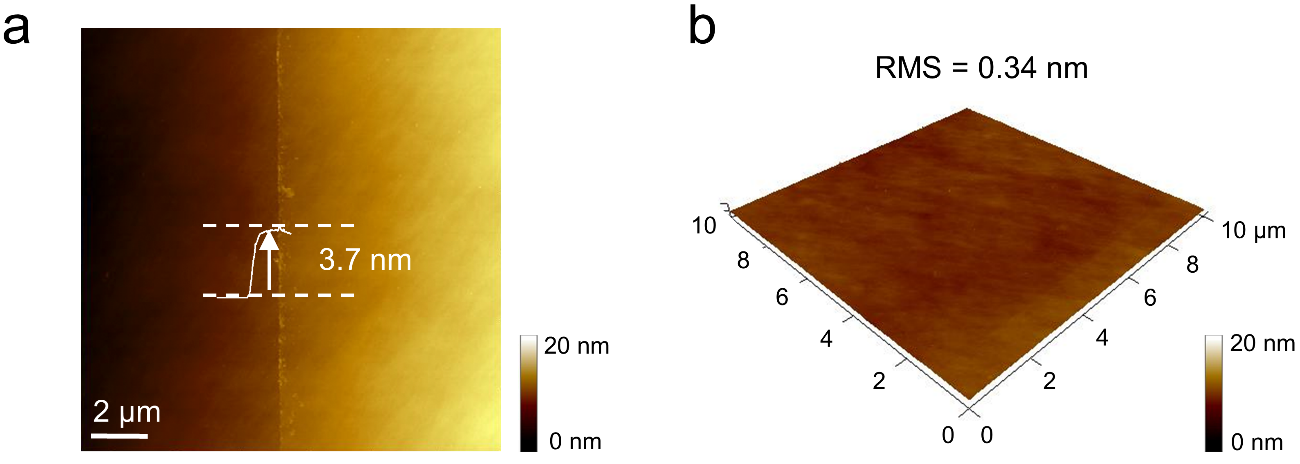


**Fig. S2** AFM images of as-exfolished GaO_x_ thin film. **a**, Height image. **b**, Surface morphology image.

As shown in Fig. S2 of the atomic force microscope (AFM) images, the thickness and root mean square (RMS) roughness of as-exfoliated GaO_x_ thin film are 3.7 nm and 0.34 nm, respectively, demonstrating the compact surface and uniform morphology.


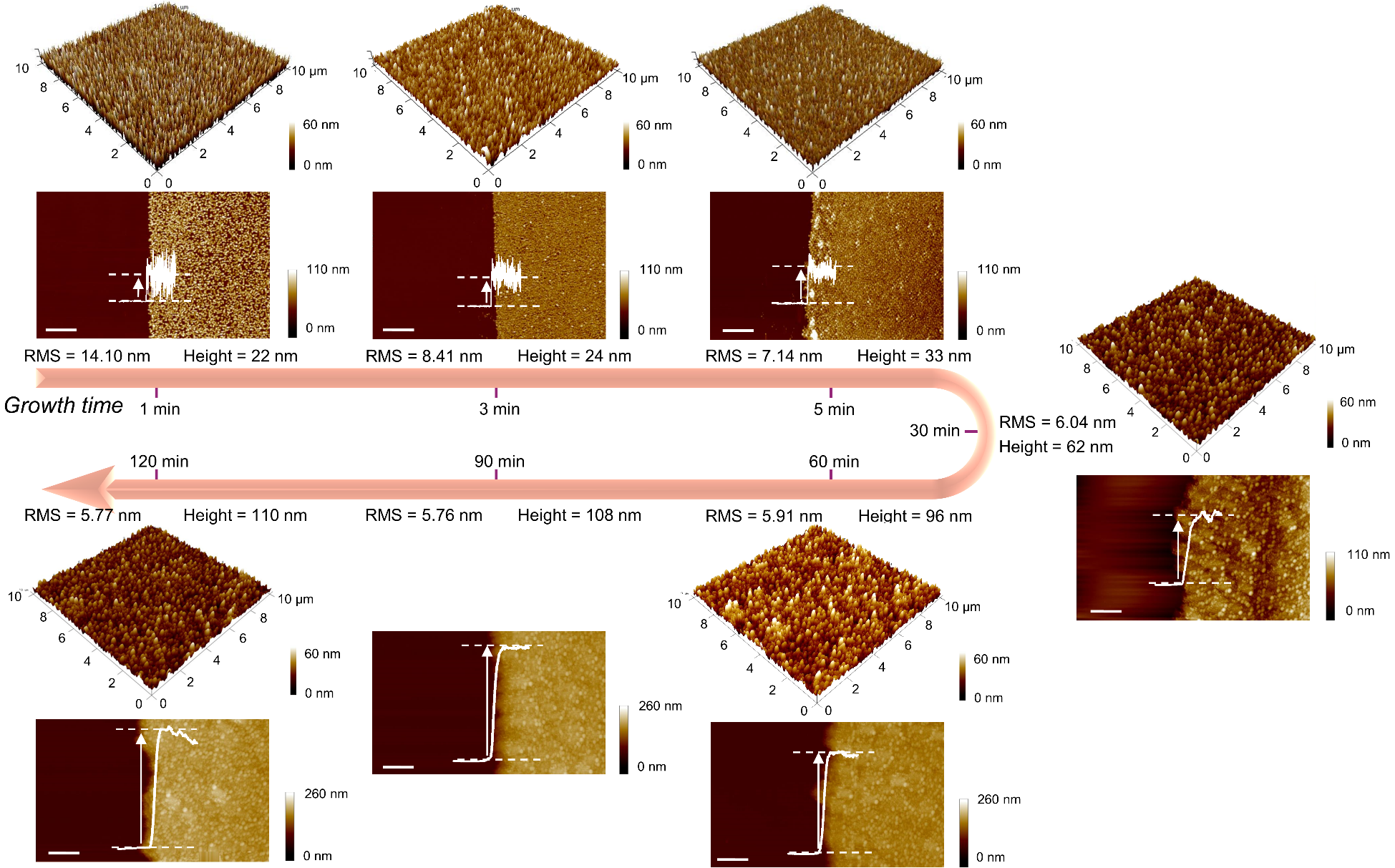


**Fig. S3** AFM images of heights and surface morphologies versus different growth durations of GaSb thin films.

As presented in Fig. S3, the thickness and RMS roughness of as-prepared GaSb films with varying durations of growth are characterized by AFM. The thickness increases from 22 nm to 108 nm, and the RMS roughness decreases from 14.1 nm to 5.76 nm with the extension of growth duration. These values remain almost unchanged after 90 min.


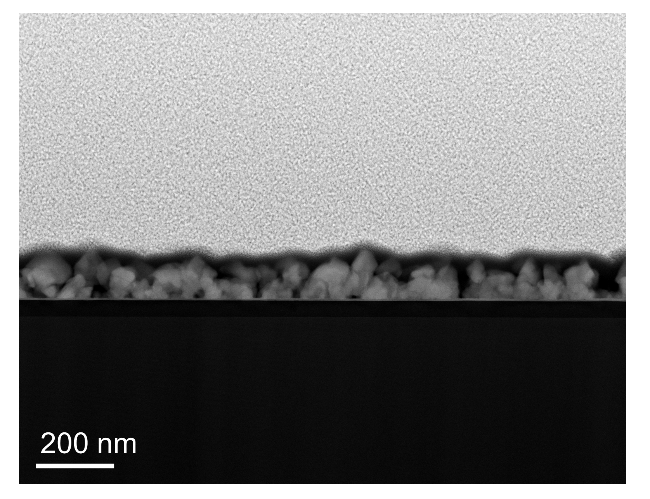


**Fig. S4** Cross-section TEM image of GaSb thin film.

As verified by the cross-section TEM image in Fig. S4, the as-prepared GaSb thin film exhibits compact and smooth surface morphology.


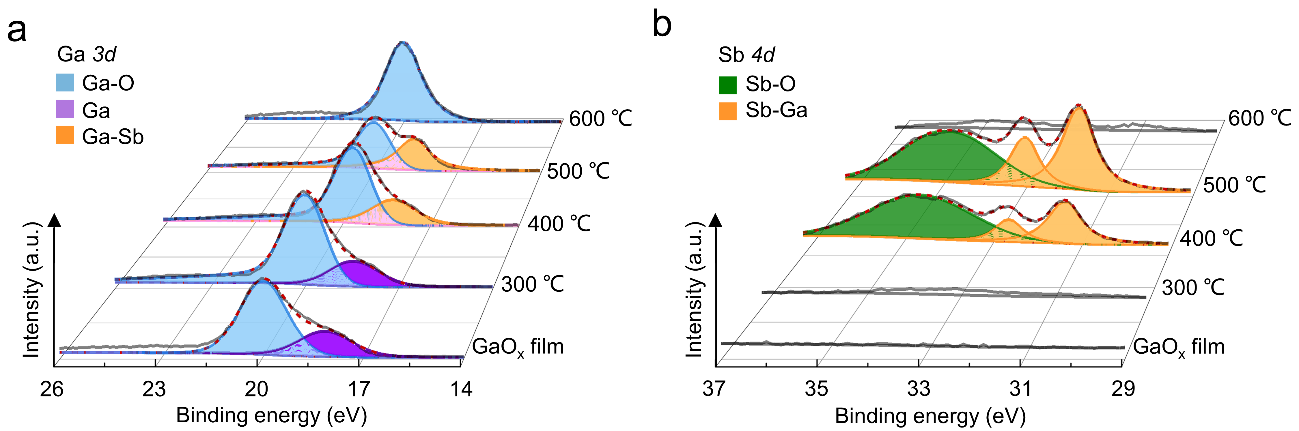


**Fig. S5** XPS spectra of Ga *3d* and Sb *4d* of as-exfoliated GaO_x_ and as-prepared GaSb films.

Fig. S5 exhibits XPS spectra of Ga *3d* and Sb *4d* of as-exfoliated GaO_x_ and as-prepared GaSb films. The primary/shoulder peaks centered at 20.0/17.9 eV are observed for as-exfoliated GaO_x_ film, corresponding to the Ga-O bond and metal Ga, respectively. This indicates that some Ga atoms are completely free in the as-exfoliated GaO_x_ film. This result resembles the film prepared at a low growth temperature of 300 °C. When the growth temperature reaches 400 °C, peaks of the Ga-Sb bond (at 18.5 eV in Ga *3d* and 31.3/32.6 eV in Sb *4d*) are observed. However, when the growth temperature reaches 600 °C, only peaks of Ga-O bond are observed. At the same time, the film prepared at 500 ℃ shows more substantial Ga-Sb peaks than that prepared at 400 ℃. All results demonstrate the optimal growth temperature of GaSb film is 500 ℃.


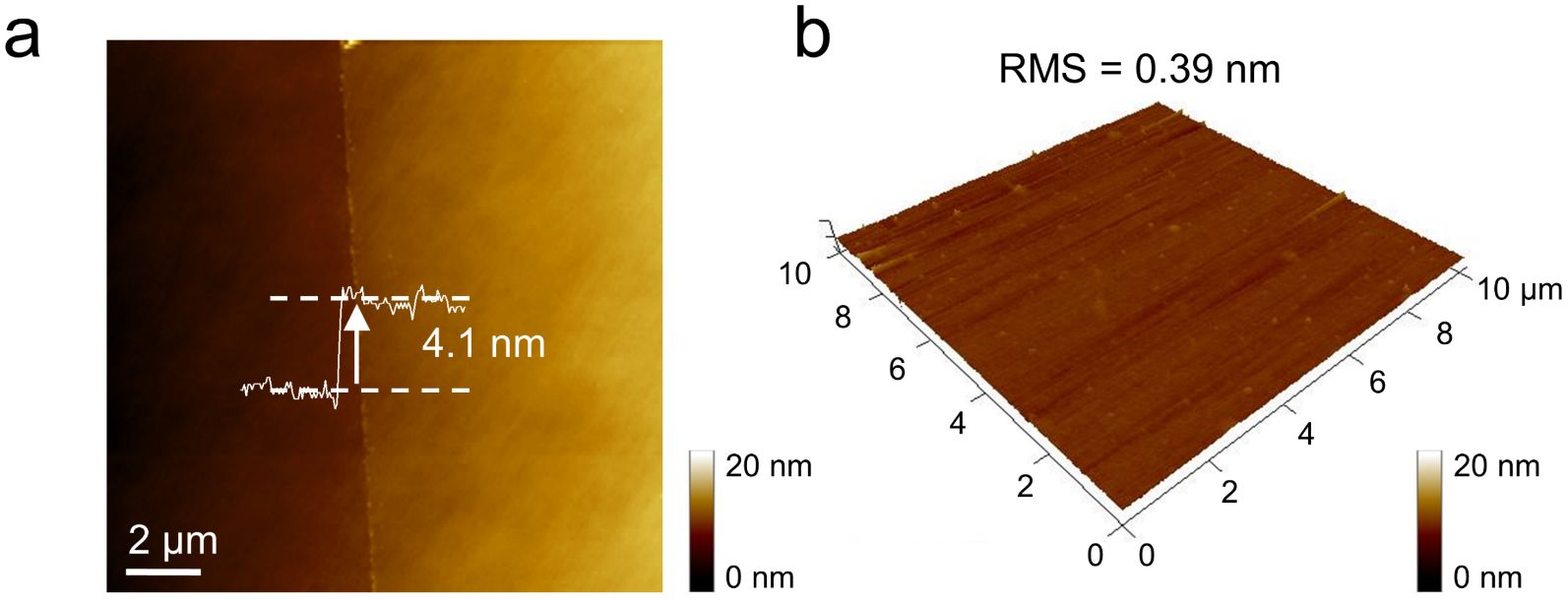


**Fig. S6** AFM images of the spin-coating GaO_x_ thin film. **a**, Height image. **b**, Surface morphology image.

Gallium nitrate hydrate (Ga(NO_3_)_3_⸱xH_2_O) (99.999%, Sigma-Aldrich) is used as gallium precursor. Exactly 399.6 mg of (Ga(NO_3_)_3_⸱xH_2_O) is separately dissolved in 10 ml DI water. After complete dissolution, the precursor solution is spun onto a 50 nm Si/SiO_2_ substrate at 3000 rpm for 20 s and annealed at 300 ℃ on a hot plate for 20 min. After that, the spin-coating GaO_x_ thin film is successfully prepared. The surface of spin-coating GaO_x_ thin film is also checked by AFM, as shown in Fig. S6. The thickness and RMS roughness are 4.1 nm and 0.39 nm, respectively, also demonstrating the compact surface and uniform morphology. Although the surface is similar to as-exfoliated GaO_x_ thin film, the spin-coating GaO_x_ thin film can not be used to the growth of GaSb thin film. This is because there is no free metal Ga in the spin-coating GaO_x_ thin film, as verified by XPS of Fig. S7.


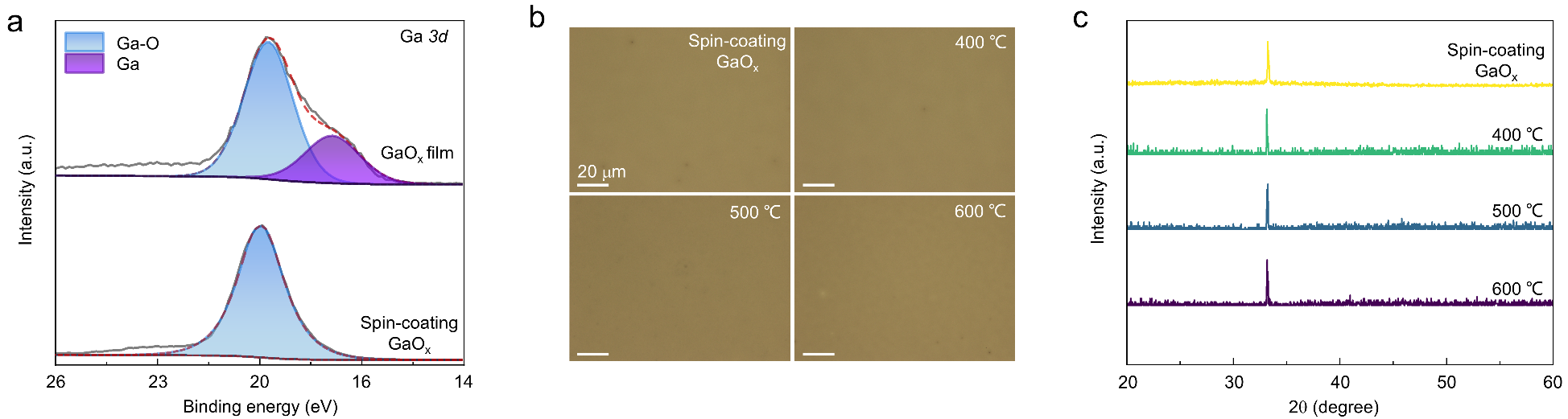


**Fig. S7 a**, XPS spectra of Ga *3d* of GaO_x_ thin film and spin-coating GaO_x_ thin film. **b**, **c**, Optical images and XRD patterns of spin-coating GaO_x_ thin film and the samples under different growth temperatures.

Spin-coating GaO_x_ film is prepared to verify whether GaSb film can be grown on its surface, as shown in Fig. S7. According to the XPS spectra of Ga *3d* in Fig. S7a, no peaks of free metal Ga are observed in the spin-coating GaO_x_ film. Figs. S7b-c exhibit the optical images and XRD patterns of spin-coating GaO_x_ film and the samples under different growth temperatures. No GaSb films are growing on the surface of the spin-coating GaO_x_ film. The results above demonstrate the crucial role of free Ga in the growth of GaSb films.


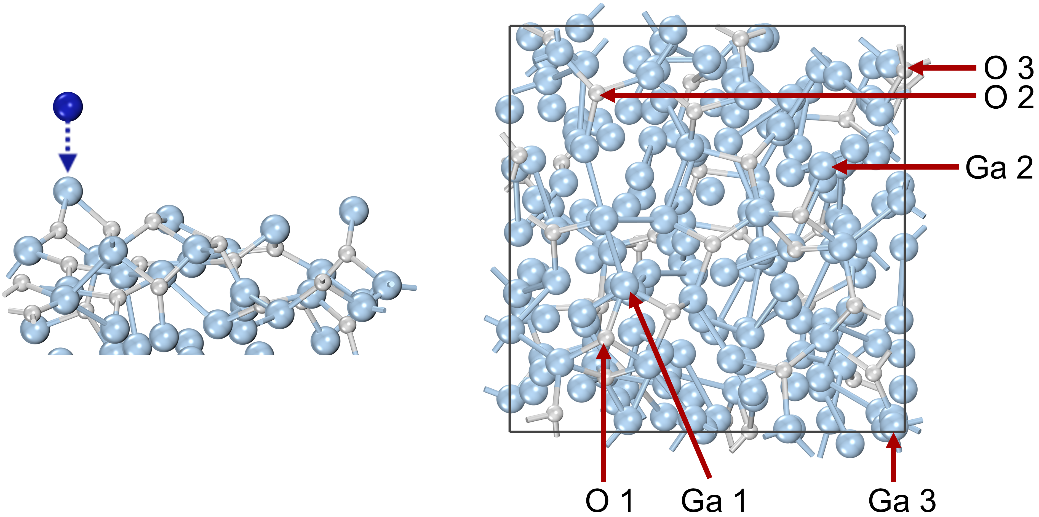


**Fig. S8** Schematic diagram of the introduced Sb atom as it gradually approaches three selected Ga or O atoms.

Fig. S8 presents the process of the introduced Sb atom as it gradually approaches three selected Ga or O atoms. The right diagram shows the position of as-selected Ga and O atoms.


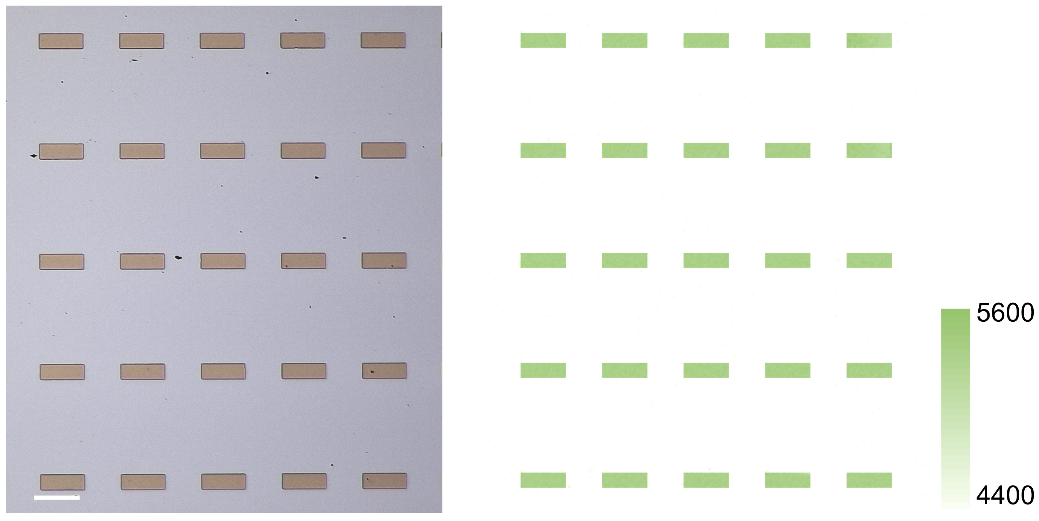


**Fig. S9** Raman mapping of patterned GaSb film array. The scale bar is 200 μm.

Fig. S9 presents the optical image and Raman mapping of the patterned GaSb film array, illustrating excellent patterning ability and large-scale uniformity of as-prepared GaSb film.


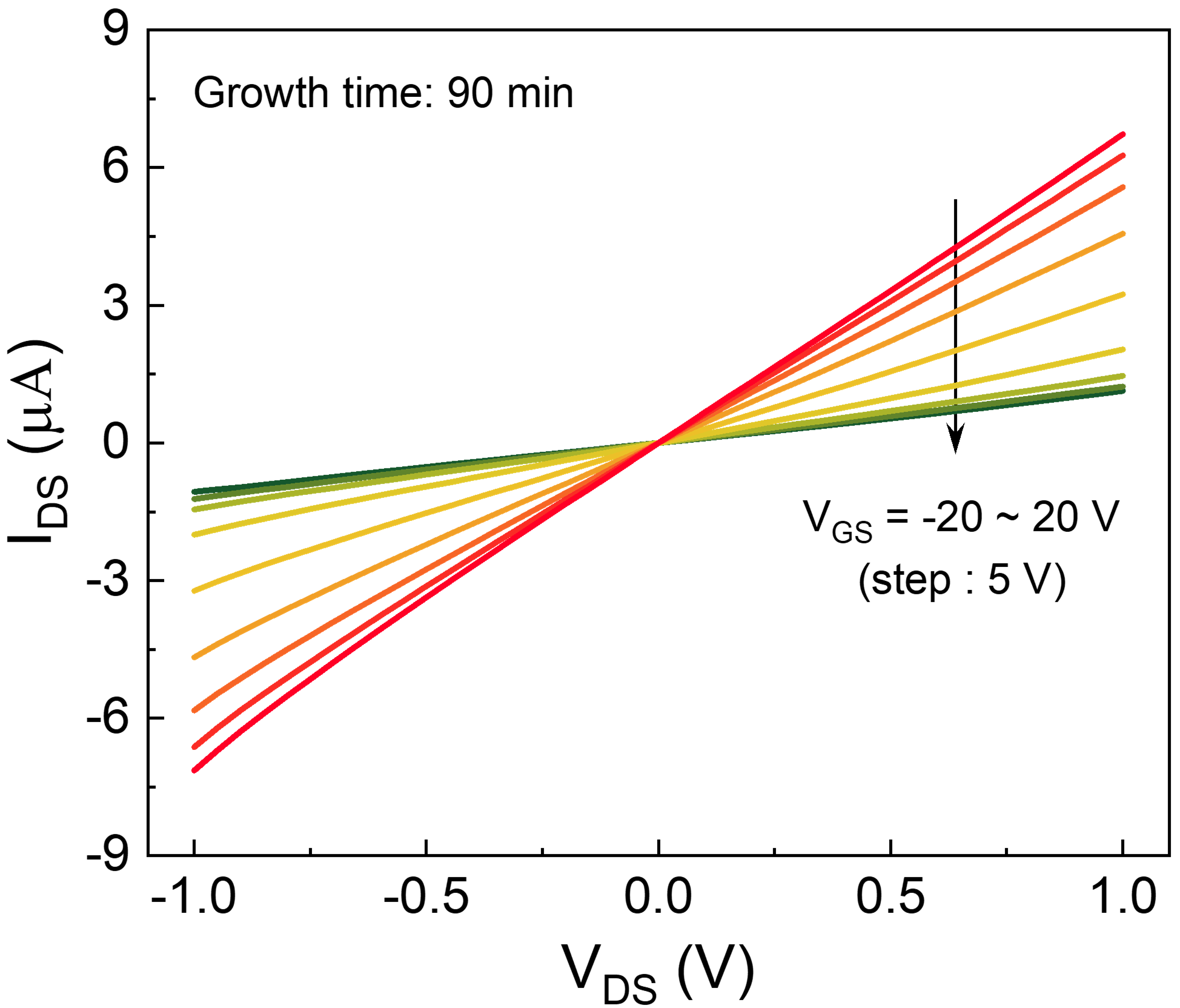


**Fig. S10** The output curves versus different V_GS_ of 90-min-grown GaSb thin film transistor.

Fig. S10 presents the output curves versus different V_GS_ of 90-min-grown GaSb film transistor, exhibiting typical p-type conducting behaviors.


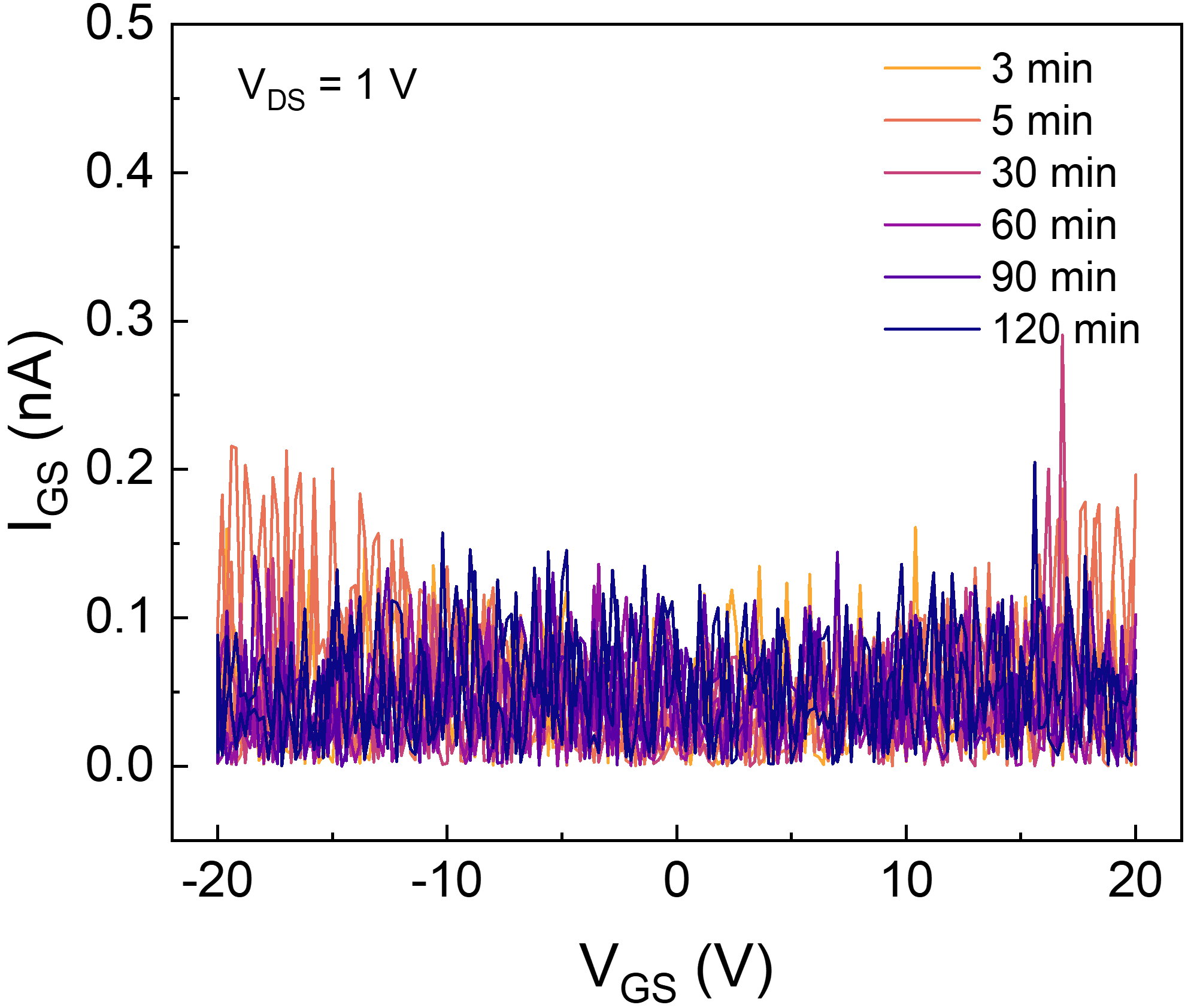


**Fig. S11** The I_GS_ curves of the as-patterned GaSb thin film transistors.


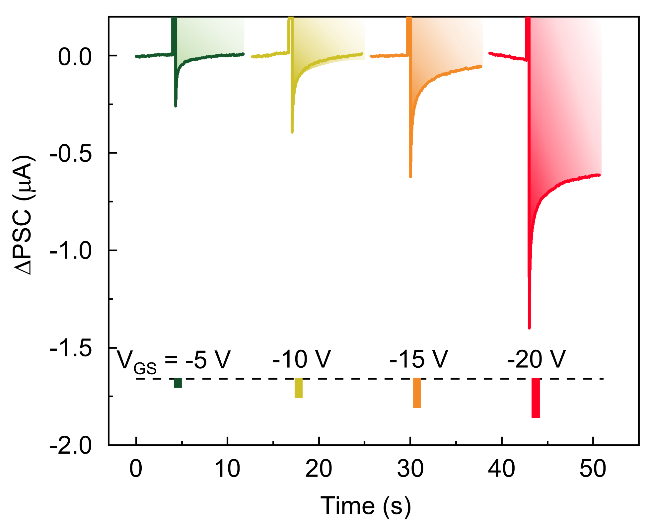


**Fig. S12** Synaptic plasticity triggered by -V_GS_ pulses (width = 100 ms).

In Fig. S12, as the V_GS_ pulses change from -5 to -20 V, ΔPSC changes from -0.26 to -1.4 μA, inferring the programmable depression behaviors of the GaSb film synaptic transistor.

**
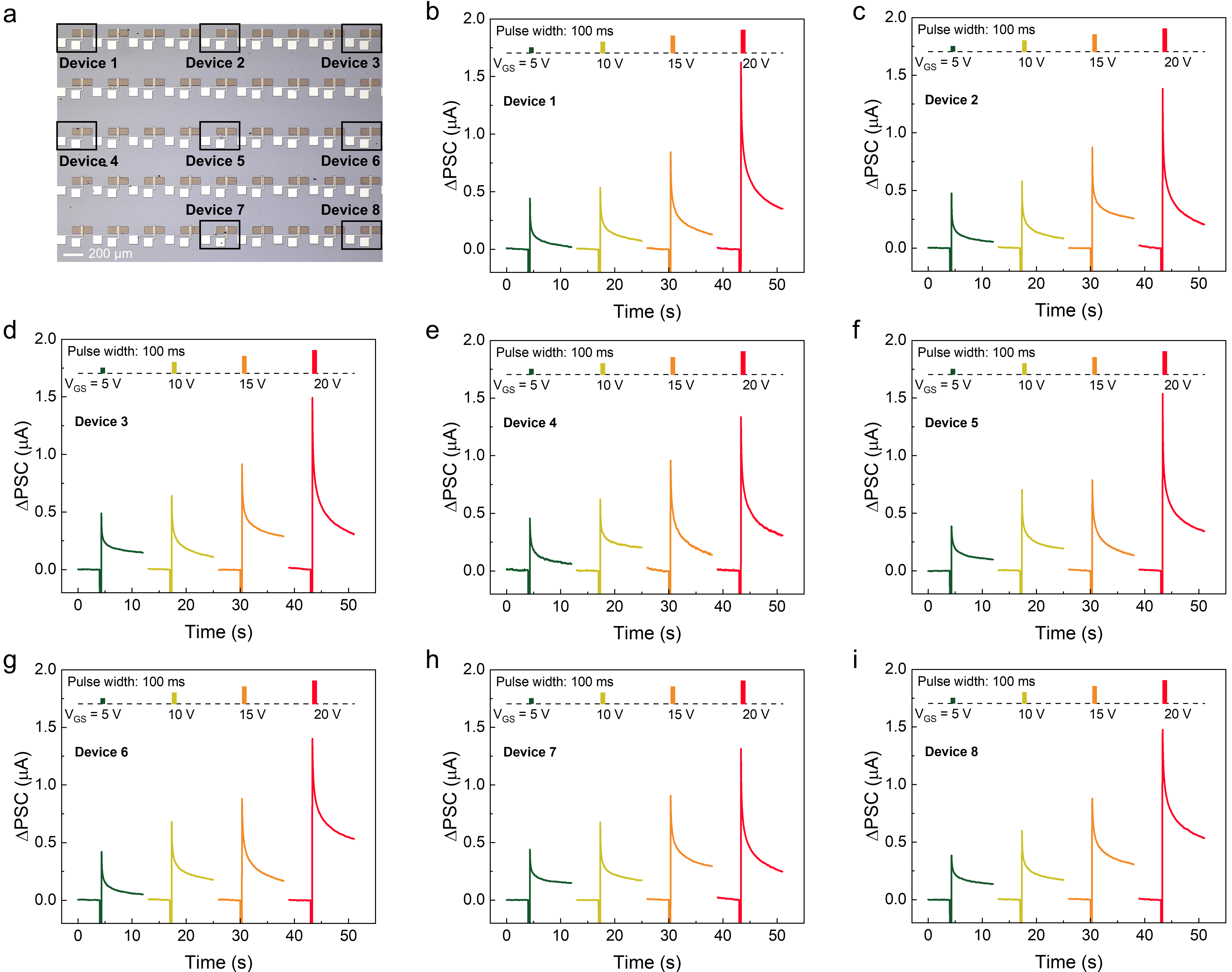
**

**Fig. S13** Synaptic plasticity of the random eight GaSb thin film synaptic transistors. **a,** The optical image of GaSb thin film synaptic transistors array. **b-i,** Positive V_GS_ pulses-triggered synaptic plasticity of eight synaptic transistors.


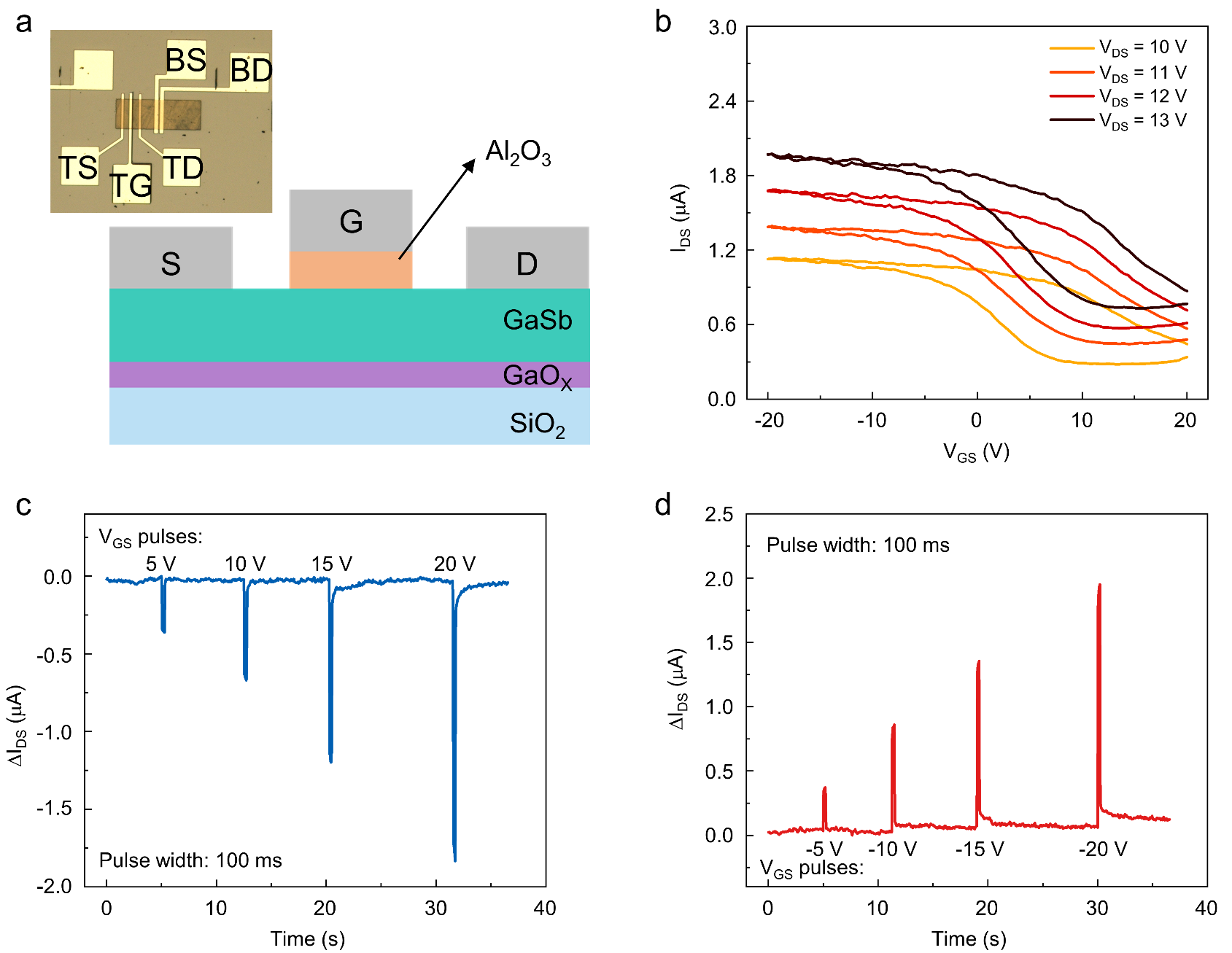


**Fig. S14** Electrical performance of top gate GaSb TFT. **a**, The optical image and schematic diagram of top-gated GaSb TFT. **b**, Transfer characteristics. **c**, **d**, The change of I_DS_ triggered by +V_GS_ and -V_GS_ pulses (width = 100 ms).

To explore the mechanism that enables synaptic behaviors in GaSb film transistors, the top-gated GaSb film transistor is constructed and studied in Fig. S14. As shown in Fig. 14a, 60 nm Al_2_O_3_ is adopted as dielectric. Compared with the back-gated film transistor, there is no amorphous GaO_x_ film between the Al_2_O_3_ and GaSb film. As expected, p-type conductive behaviors are observed in the top-gated GaSb film transistor in Fig. S14b. When positive or negative V_GS_ pulses are applied, no obvious synaptic behaviors are observed in Figs. S14c-d. The amorphous GaO_x_ film enables the synaptic behaviors of the GaSb film transistor.


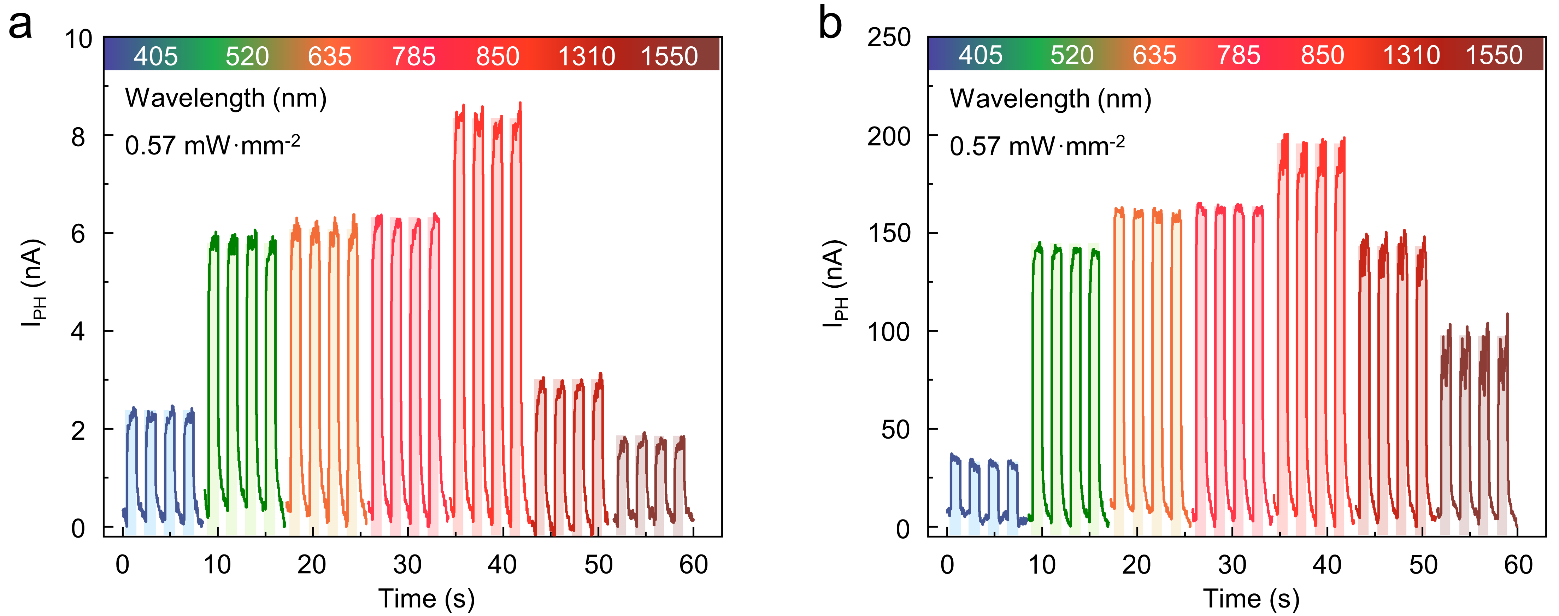


**Fig. S15 a**, **b**, Wavelength-dependent broadband photodetection behaviors of GaSb thin films with 5 and 120 minutes growth duration. The laser intensity is 0.57mW⸱mm^-2^.

Fig. S15 exhibits the excellent broadband photodetection behaviors of GaSb films with growth durations of 5 and 120 minutes.


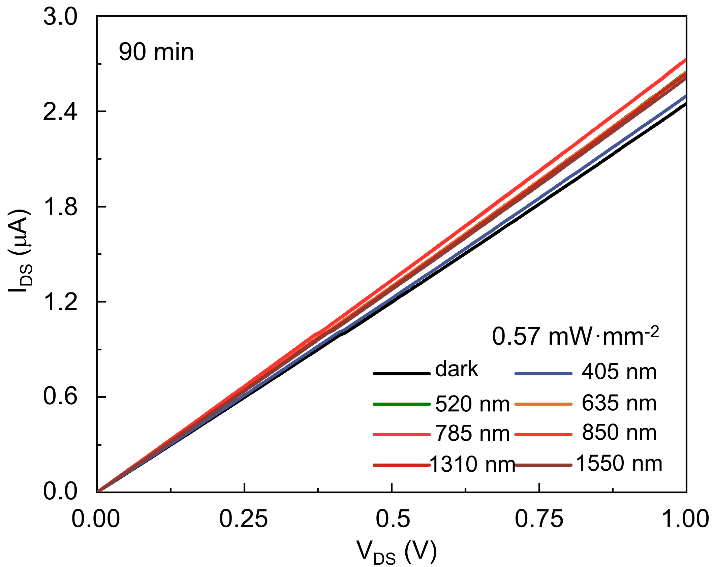


**Fig. S16** Wavelength-dependent static photodetection behaviors of GaSb film with a growth duration of 90 minutes. The laser intensity is 0.57mW⸱mm^-2^.

Fig. S16 shows the excellent static broadband photodetection performance of GaSb film with a growth duration of 90 min.


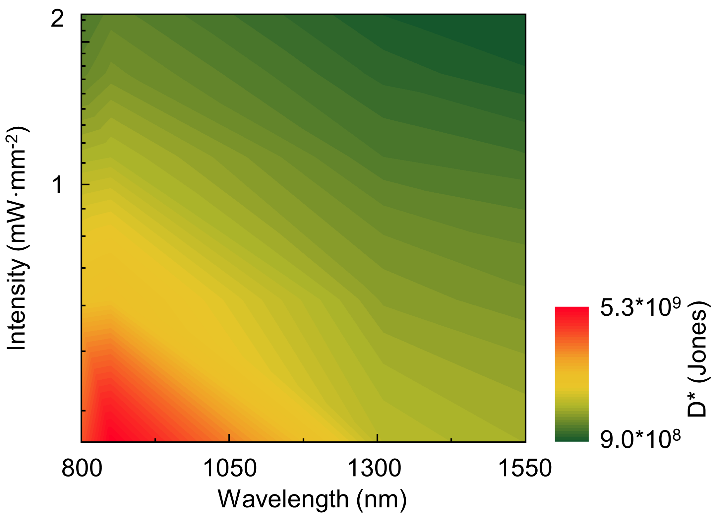


**Fig. S17** Two-dimensional contour map of D* versus different wavelengths and laser intensity.

Under different wavelengths and light intensities, D* of GaSb film grown by 90 min is studied in Fig. S17. The maximum D* can be as high as 5.3×10^9^ Jones under the illumination of an 850 nm laser with an intensity of 0.28 mW⸱mm^-2^.


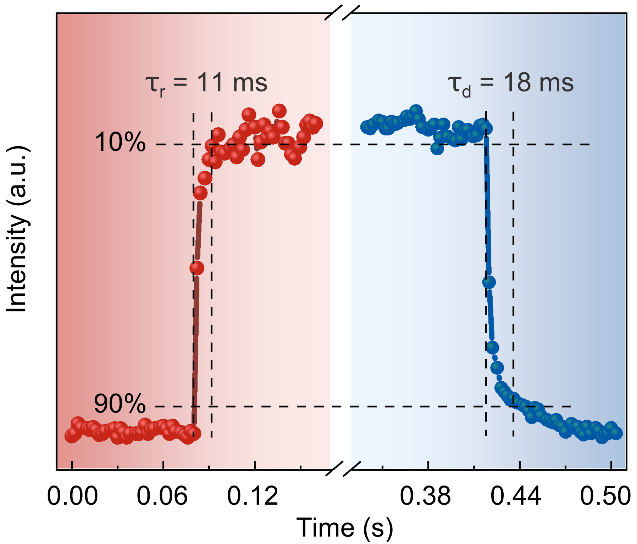


**Fig. S18** The temporal photoresponse of GaSb film.

The temporal photoresponse of GaSb film is shown in Fig. S18. Benefiting from excellent photodetection characteristics, the as-constructed GaSb film photodetector exhibits τ_r_ of 11 ms and τ_d_ of 18 ms.


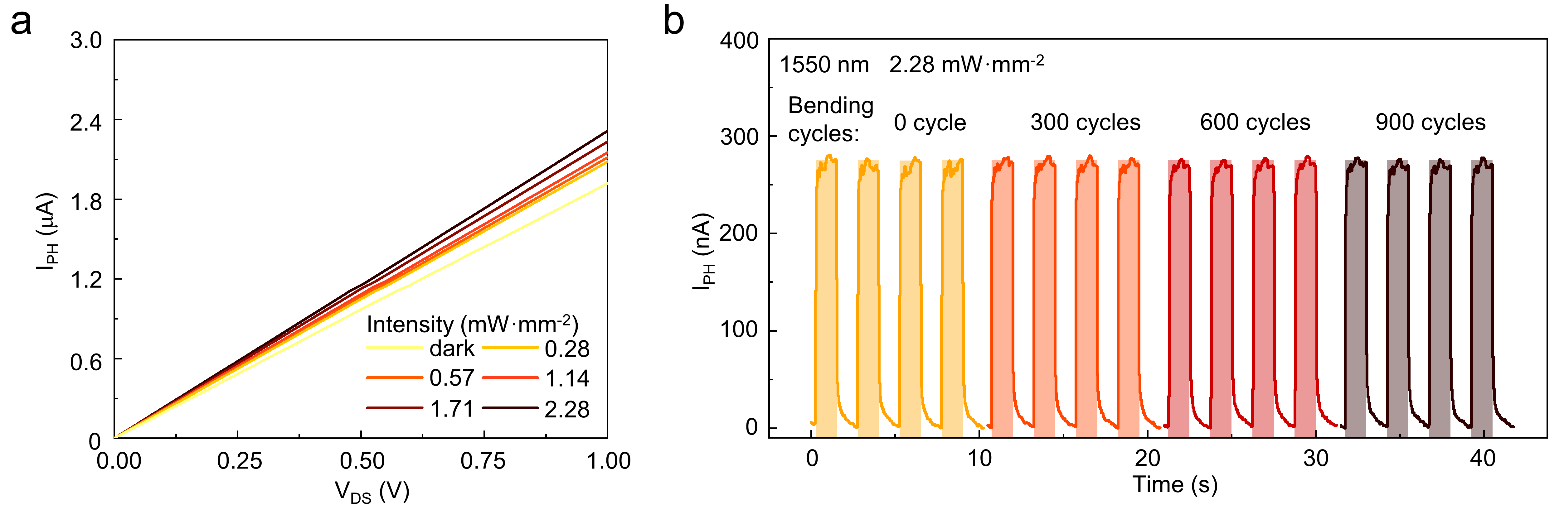


**Fig. S19 a**, Output characteristics under the illumination of 1550 nm laser. **b**, Time-resolved photoresponse of as-fabricated flexible photodetector after different bending radius.

As-fabricated GaSb film flexible photodetector exhibits typical ohmic contact and excellent static broadband photodetection performance in Fig. S19a. After 900 bending-recovering cycles, there is almost no apparent attenuation of I_PH_ in Fig. S19b, exhibiting good bendability and flexibility.


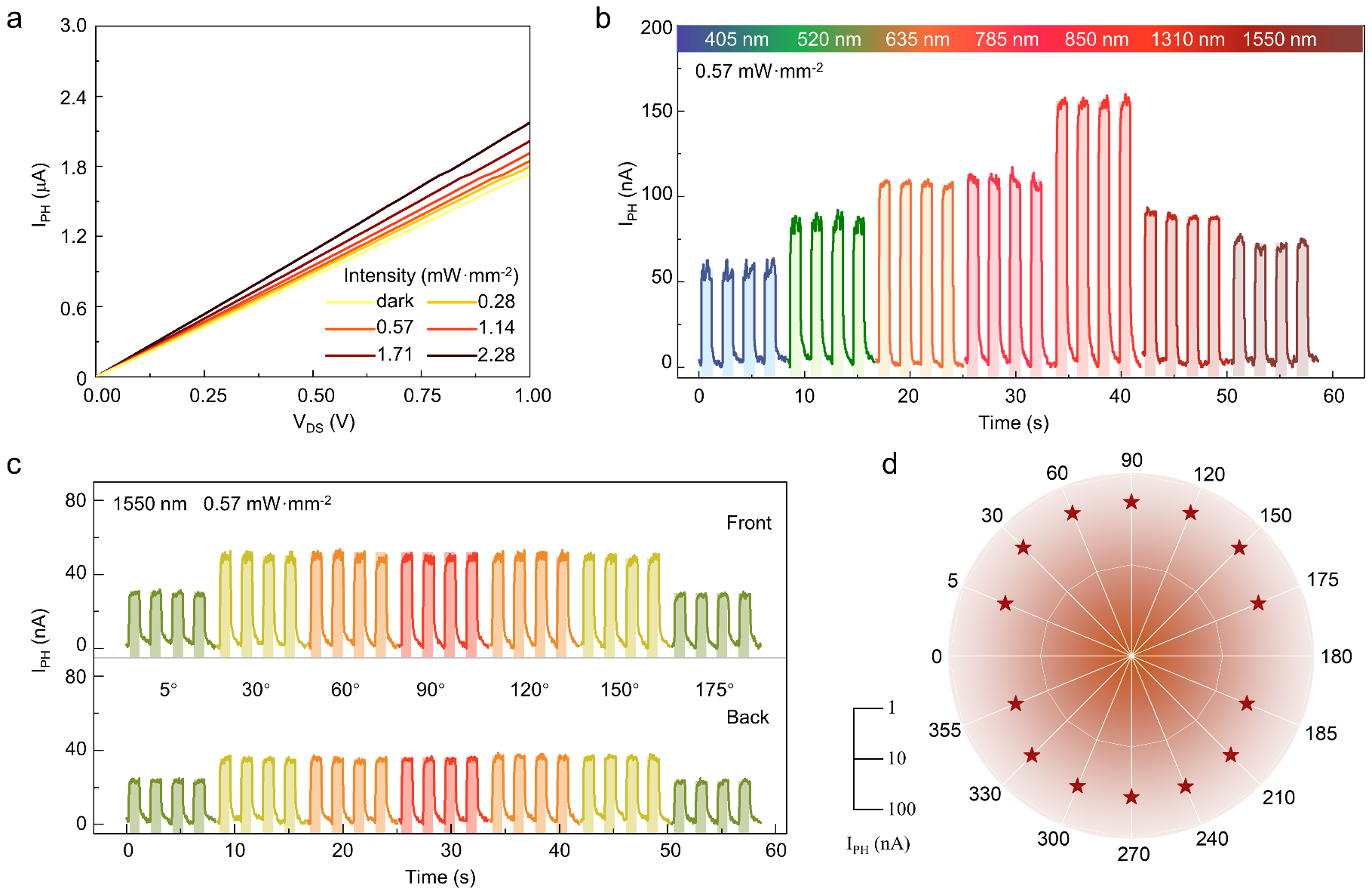


**Fig. S20** Omnidirectional photodetection behaviors of GaSb film on a glass substrate. **a**, Output characteristics under the illumination of 1550 nm laser. **b**, Wavelength-dependent broadband photoresponse. **c**, **d**, Omnidirectional photodetection behaviors.

Benefiting from the weak substrate dependence of the induced fit growth method, GaSb film can be grown on rigid, transparent glass, promising an omnidirectional photodetector. Typical ohmic contact is demonstrated in Fig. S20a. As-prepared GaSb film shows as-expected broadband photodetection behaviors in Fig. S20b. When the laser irradiates the photodetector in omnidirectional directions, the variation of I_PH_ is recorded and shown in Figs. S20c-d. A stable photoresponse can be observed even at an incident angle of 5°, and the I_PH_ can keep over 61% compared to that at an incident angle of 90°, exhibiting excellent omnidirectional photodetection performance.


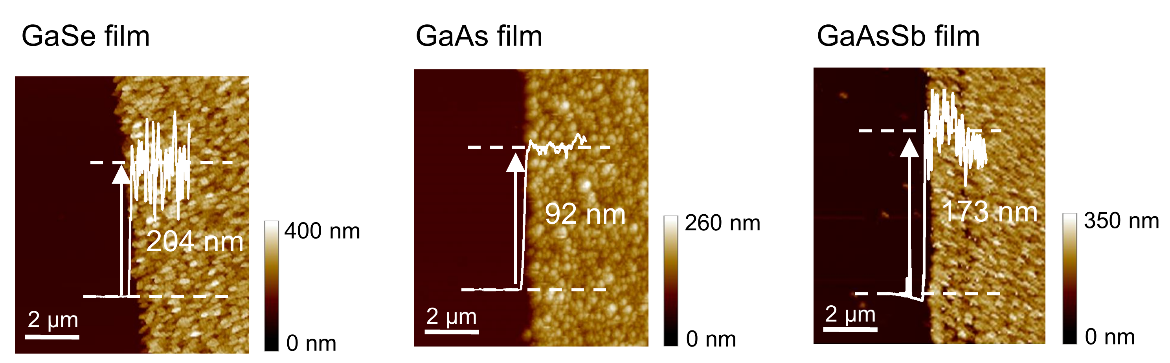


**Fig. S21** AFM images of GaSe, GaAs and GaAsSb films.

The thicknesses of GaSe, GaAs, and GaAsSb films are characterized by AFM in Fig. S21, exhibiting 204 nm, 92 nm, and 173 nm, respectively.


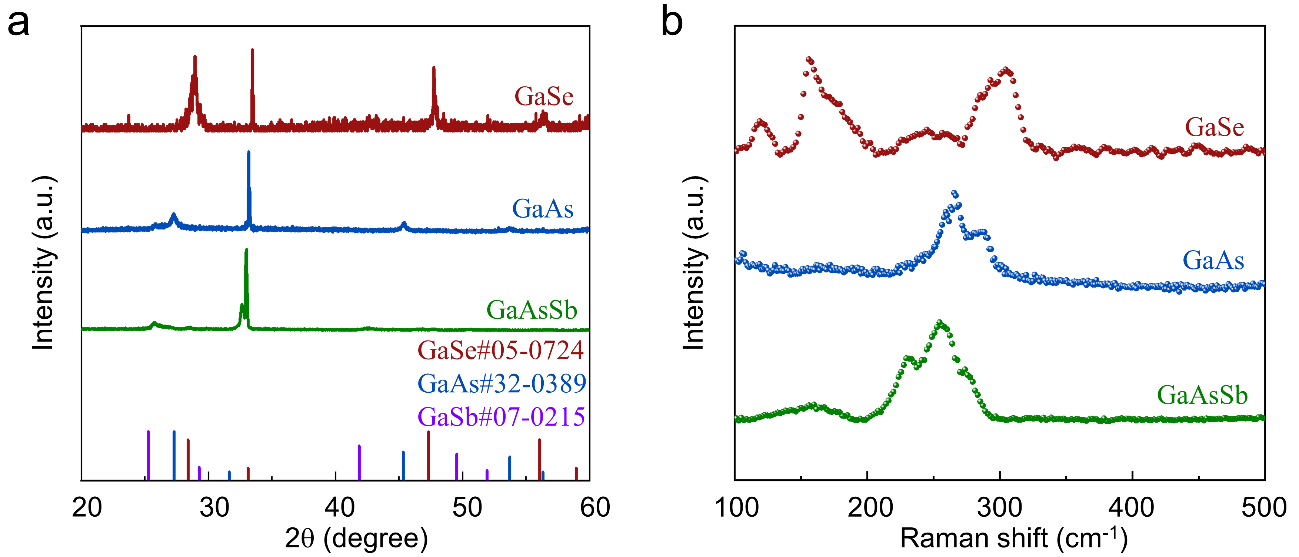


**Fig. S22 a**, **b**, XRD patterns and Raman spectra of GaSe, GaAs, and GaAsSb films.

According to the XRD patterns and Raman spectra in Fig. S22, as-prepared GaSe, GaAs, and GaAsSb films exhibit good crystallinity.


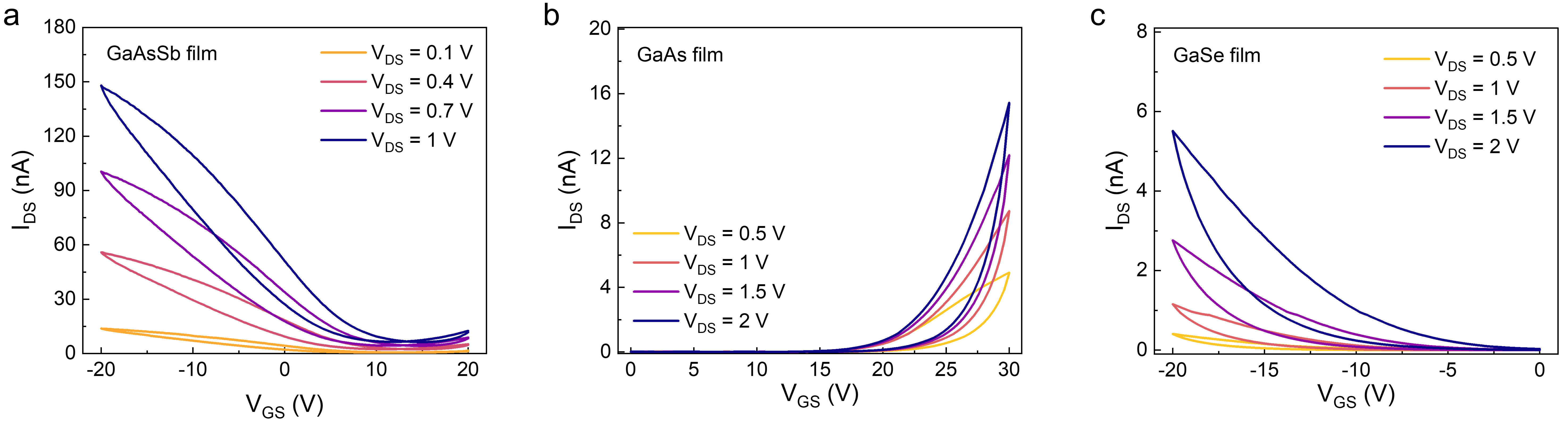


**Fig. S23** Transfer characteristics of GaAsSb (a), GaAs (b) and GaSe (c) thin films transistors.

As shown in Fig. S23, the as-prepared GaAsSb, GaAs, and GaSe thin films exhibit p-type, n-type and p-type conducting behaviors, respectively.


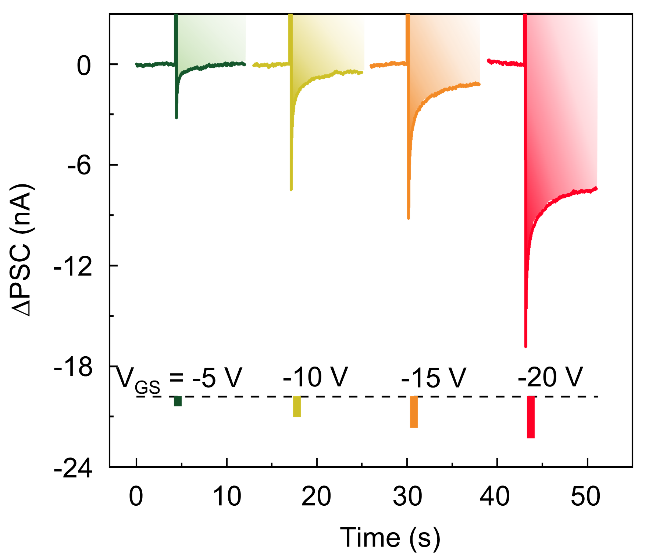


**Fig. S24** Synaptic plasticity triggered by -V_GS_ pulses (width = 100 ms) of GaAsSb synaptic transistor.

In Fig. S24, as the V_GS_ pulses change from -5 to -20 V, ΔPSC changes from -3.2 to -16.8 nA, inferring the programmable depression behaviors of the GaAsSb film synaptic transistor.


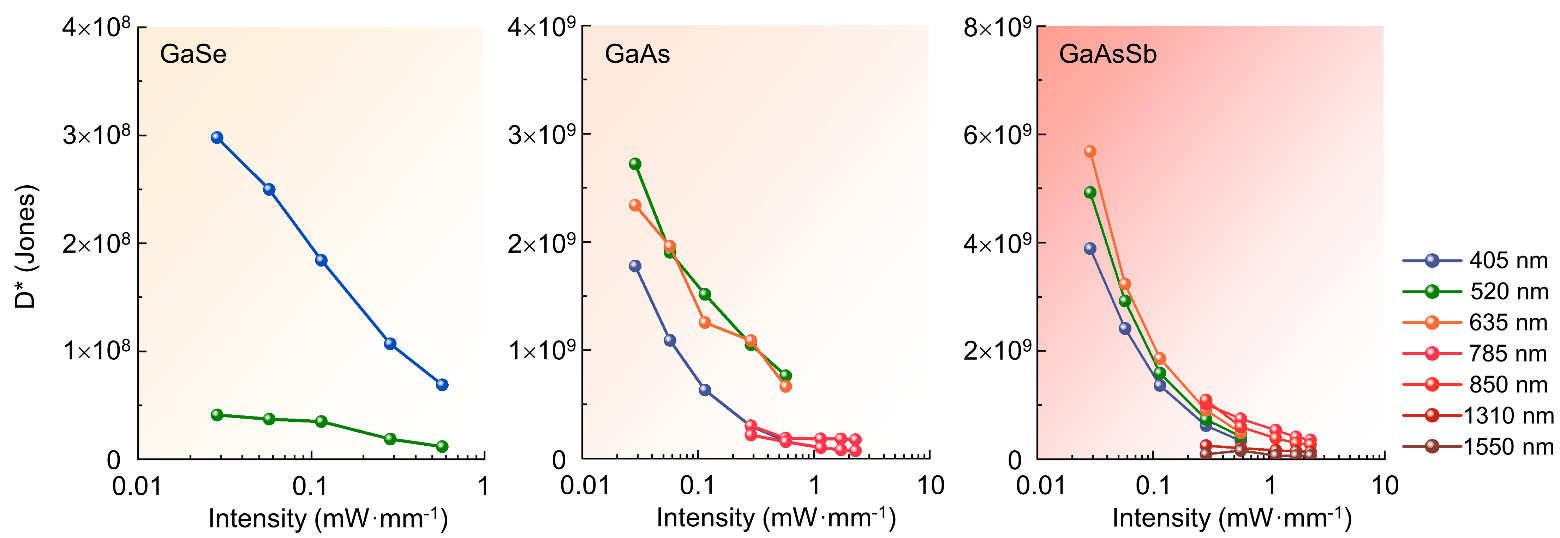


**Fig. S25** Wavelength-dependent D^*^ of GaSe, GaAs, and GaAsSb films.

Under illumination of different wavelengths and different light intensities, D* of GaSe, GaAs, and GaAsSb films are studied in Fig. S25.

**References**

1. Zaumseil, P. High-resolution characterization of the forbidden Si 200 and Si 222 reflections. *J. Appl. Cryst.* **48**, 528-532 (2015).

2. Hesse, D., Lee, S.K. & Gösele, U., Microstructure of (104)-oriented Bi_3.25_La_0.75_Ti_3_O_12_ and Bi_3.54_Nd_0.46_Ti_3_O_12_ ferroelectric thin films on multiply twinned SrRuO_3_/Pt(111) electrodes on YSZ(100)-buffered Si(100). *phys. stat. sol. (a)* **202**, 2287-2298 (2005).

3. Zhao, L. et al. Lithium Niobate microtubes within ordered macroporous silicon by templated thermolysis of a single source precursor. *Chem. Mater.* **17**, 3-5 (2005)
